# In Vitro Biotransformation of Ziziphi Spinosae Semen Saponins by Gut Microbiota from Healthy and Insomniac Groups

**DOI:** 10.3390/ijms26094011

**Published:** 2025-04-24

**Authors:** Xiaofang Cui, Shengmei Zhang, Ling He, Huizhu Duan, Yujun Xie, Xiangping Pei, Yan Yan, Chenhui Du

**Affiliations:** 1 School of Chinese Materia Medica, Shanxi University of Chinese Medicine, Taiyuan 030619, China; xiaofangcui2025@163.com (X.C.); 13145263888@163.com (S.Z.); 13994154651@163.com (L.H.); duanhz0506@163.com (H.D.); yujun_xie@163.com (Y.X.); peixp69@163.com (X.P.); 2 Modern Research Center for Traditional Chinese Medicine, Shanxi University, Taiyuan 030006, China

**Keywords:** biotransformation, human gut microbiota, insomnia, UPLC-Q-Orbitrap-MS, Ziziphi Spinosae Semen saponins

## Abstract

Ziziphi Spinosae Semen saponins (ZSSS) show sedative–hypnotic activity but have very low bioavailability, potentially due to their conversion into bioactive metabolites by gut microbiota. In this study, the biotransformation of ZSSS by gut microbiota from healthy humans and patients with insomnia in vitro was analyzed. A total of 21 prototype compounds and 49 metabolites were identified using UHPLC-Q-Orbitrap-MS. Deglycosylation, deoxygenation, dehydration, and deacylation were detected in both healthy individuals and insomniacs. However, oxidation and hydrogenation were uniquely observed in insomniacs. ZSSS can enhance beneficial bacteria, such as *Veillonella*, *Dialister*, and *Bacteroides.* ZSSS can promote the synthesis of short-chain fatty acids (SCFAs), especially acetic acid, propionic acid, and butyric acid. Furthermore, it was found that the sedative–hypnotic activity of ZSSS was enhanced after biotransformation, as determined by a sodium pentobarbital-induced sleeping test (SPST), open-field behavior test (OFBT), and molecular docking experiment (MDE). These results collectively offer valuable insight into the mechanism of action of ZSSS.

## 1. Introduction

Ziziphi Spinosae Semen (ZSS), a seed of *Ziziphus jujuba* Mill. var. *spinosa* Hu ex H. F. Chou, has been extensively used to treat insomnia and anxiety for over 2000 years in clinical practice [1]. ZSSS, also called jujubosides or triterpenoid glycosides, are considered the major active compounds of ZSS. ZSSS can be divided into tetracyclic triterpenoids saponins (TTSs) and pentacyclic triterpenoids saponins (PTSs), based on their structural skeletons. The chemical and pharmacological diversity and potential structure–activity relationships of ZSSS have been reviewed and highlighted. As the most abundant compounds, ZSSS have been demonstrated to possess various activities, including improving sleep quality, anti-anxiety, anti-depression, and improving Alzheimer’s disease [2]. However, the oral bioavailabilities of jujuboside A (JuA) and jujuboside B (JuB) in rats were only 1.3 and 3.6%, respectively [3,4]. The low bioavailability of triterpene saponins by oral administration has become a bottleneck in the development of ZSSS, despite increasing attention in recent years.

The gut microbiome, a complex ecosystem of nearly 10^14^ microorganisms, directly or indirectly alter the pharmacokinetics and pharmacological activity of drugs [5], especially oral herbs such as ZSS. Conversely, if the composition or metabolites of the intestinal flora are abnormal, it may, in turn, lead to an imbalance in the flora ecology [6]. Characterization of metabolites and metabolic profiling of ZSSS in the intestinal tract is a vital method for elucidating the active components of ZSSS. However, research on the biotransformation of ZSSS in human gut microbiota is lacking because of the shortage of reference compounds and the complex structures of ZSSS extract.

According to reported studies, saponins are easily decomposed into sapogenins during intestinal metabolism for efficient absorption. JuA and JuB, which are the most important active ingredients in ZSSS, can be biotransformed into a series of secondary saponins, such as Zizyphus saponin II (Ziz II) and Bacopaside IV, as well as jujubogenin, through the step-by-step removal of their sugar residues by gut microbiota [7]. Furthermore, the metabolites, including JuB and jujubogenin, exhibit significant effects on the expression and activation of γ-amino-butyric acid A (GABA_A_) receptors [8]. Jujubogenin was suggested to be the effective constituent in ZSS for exerting the sedative function via GABA_A_ receptor [9]. These data imply that the bioconversion of ZSSS mediated by gut microbiota could lead to different responses in its sedative–hypnotic effects. Existing studies primarily focus on the metabolic processes of JuA and JuB; however, these studies on single components fail to elucidate the comprehensive metabolic process of ZSSS.

It was demonstrated that the composition, diversity, and metabolic function of the gut microbiota differ significantly between healthy humans and patients with insomnia [10]. The enzymes secreted by various gut microorganisms differ, leading to metabolic differences in saponins. The biotransformation of ZSSS might vary considerably because of variations in gut microbiota between healthy individuals and patients with insomnia, which could alter the sedative–hypnotic properties of ZSSS. Therefore, it is meaningful to investigate the bioconversion of ZSSS mediated by gut microbiota derived from healthy volunteers and patients with insomnia.

In this work, a biotransformation study of ZSSS using the gut microbiota of healthy humans and those with insomnia was conducted in vitro. The biotransformation pathways of gut microbiota between healthy individuals and patients with insomnia were explored by comparing the differences in metabolic transformation. The changes in gut microbiota structure and short-chain fatty acids (SCFAs) content during in vitro fermentation were studied, and the correlation between them was analyzed. The sedative–hypnotic activity of ZSSS before and after biotransformation was also compared using SPST and MED. This study aims to provide scientific mechanistic evidence to support the popular use of ZSSS in the management of sleep disorders.

## 2. Results and Discussion

### 2.1. Identification Compounds in ZSSS

**Figure 1:**
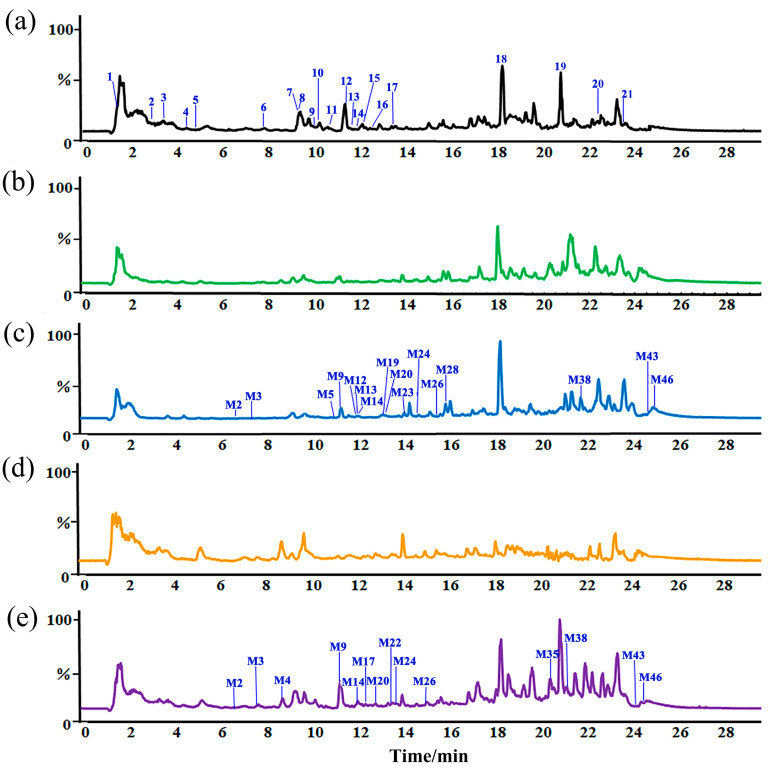
Total ion chromatograms (TICs) in the negative mode: ZSSS and general anaerobic medium (GAM) (**a**); CN group (**b**); N-48 h group (**c**); CI group (**d**); I-48 h group (**e**).

To characterize the chemical compounds of ZSSS, a UHPLC-Q-Orbitrap-MS method using the full mass/dd-MS^2^ (Top5) scan mode was developed, and the total ion chromatograms (TICs) are shown in Figure 1a and Figure S4a. A total of 21 saponins, including 17 tetracyclic triterpenoid saponins (TTSs) and 4 pentacyclic triterpenoid saponins (PTSs), were identified in ZSSS. The TTSs, including protojujuboside B (PJuB, 2), JuA (7), JuB (12), and jujuboside B_1_ (JuB_1_, 15), as well as PTSs, including ceanothic acid (CA, 18), alphitolic acid (AA, 19), betulinic acid (BA, 20), and betulin (BE, 21), were identified by comparison with the reference standards (Table S1). The retained compounds were tentatively characterized by comparing their exact molecular weights and MS^2^ fragment ions with those reported in the literature [11,12,13]. As shown in Figure 1a and Table S1, the TTSs contained eight jujuboside saponins (JS), four C17-side-chain-varied jujubogenin-type saponins (C17-VJS), and five C16-keto jujubogenin-type saponins (C16-KJS) in ZSSS. A diagram of the rapid identification of triterpenoids by UPLC-Q-Orbitrap-MS is shown in Figure S1.

For instance, the characteristic ions for JS were [M − H]^−^ or [M + HCOO]^−^ at *m*/*z* 749.45, 603.39, and 471.35 and [M + H]^+^ or [M + Na]^+^ at *m*/*z* 733.45, 587.40, and 455.35. For C17-VJS, the characteristic ions were [M − H]^−^ or [M + HCOO]^−^ at *m*/*z* 749.45 and 603.39 and [M + H]^+^ at *m*/*z* 587.40, 455.35, and 437.34. For C16-KJS, the characteristic ions were [M − H]^−^ or [M + HCOO]^−^ at *m*/*z* 919.45, 787.41, and 479.30 and [M + H]^+^ at *m*/*z* 491.37. Their fragmentation pathways are shown in Figure S2a–c.

ZSSS contains many sugar chains, with sugars generally linked to the C-3 position of the saponin nucleus. These sugars include rhamnose (Rha), xylose (Xyl), glucose (Glc), arabinose (Ara), and fucose (Fuc). The loss of 162 Da corresponds to the removal of Glc, while losses of 146 Da and 132 Da correspond to the removal of Rha or Fuc and Ara or Xyl, respectively.

In addition, the low-mass region in the negative ion mode exhibits a rich variety of fragment ions. Specifically, *m*/*z* 179.05, 161.04, 143.03, 131.03, 119.03, 113.02, and 101.02 correspond to the presence of Glc; *m*/*z* 145.04, 103.03, and 97.02 indicate the presence of Rha or Fuc; *m*/*z* 101.02 and 89.02 suggest the presence of either Ara or Xyl; *m*/*z* 191.05 and 179.05 point to the presence of Glc-Ara/Xyl composition; and *m*/*z* 221.06 indicates the presence of a Glc-Glc composition. This method allows for the inference of the number, position, composition, and linkage order of sugars. The fragmentation pathways of the glucose residue ion are shown in Figure S2d.

### 2.2. Metabolic Differences of ZSSS in Gut Microbiota from Normal Patients and Humans with Insomnia

The metabolic differences in ZSSS in normal and insomnia human gut microbiota were investigated. All incubated samples were analyzed using UHPLC-Q-Orbitrap-MS for metabolite identification. As shown in Figure 1b–e and Figure S4b–e, a total of 49 metabolites were characterized (Table S2 and Figure S3), including six prototype components and 43 metabolic transformation products of ZSSS in the N and I groups. Specifically, 43 metabolites were identified in the N group and 46 in the I group. Moreover, 39 common metabolites, primarily undergoing deglycosylation reactions, were observed in both the N and I groups (Table S3). Notably, three unique metabolites, such as JuB + C_5_H_10_O_5_ (M7), BE − H_2_O (M38), and CA − COOH (M45), were found exclusively in the N group, while six unique metabolites, including jujuboside G + 2H − O (JuG + 2H − O, M4), JuB + O (M5), jujuboside II – Rha (JuII – Rha, M13), jujuboside III – Rha (JuIII – Rha, M21), BA + H − 2O (M39), and BE − CH_2_O (M48), were identified in only the I group.

For JS, the relative contents of JuA (7), jujuboside C (JuC, 8), jujuboside A_1_ (JuA_1_, 9), jujuboside I (JuI, 13), and acetyljujuboside B (AcJuB) (17) were reduced at 6 h. Notably, AcJuB (17) was completely degraded at 48 h, whereas the other compounds were fully degraded by 24 h. In contrast, the levels of JuB (12), JuB_1_ (15), and Ziz II (14) increased at 6 h and 12 h, respectively (Table S6). These observations can be explained by the sequential transformations of AcJuB (17); the loss of an acetyl group generates JuB (12), which subsequently loses a Xyl to form Ziz II (14). Additionally, JuB_1_ (15) is derived from JuA_1_ (9) through the removal of a Glc. Furthermore, JuA (7) can also yield JuB (12) by losing a Glc. Similarly, JuC (8) sequentially loses two Glc, first forming JuI (13) and then Ziz II (14).

**Figure 2:**
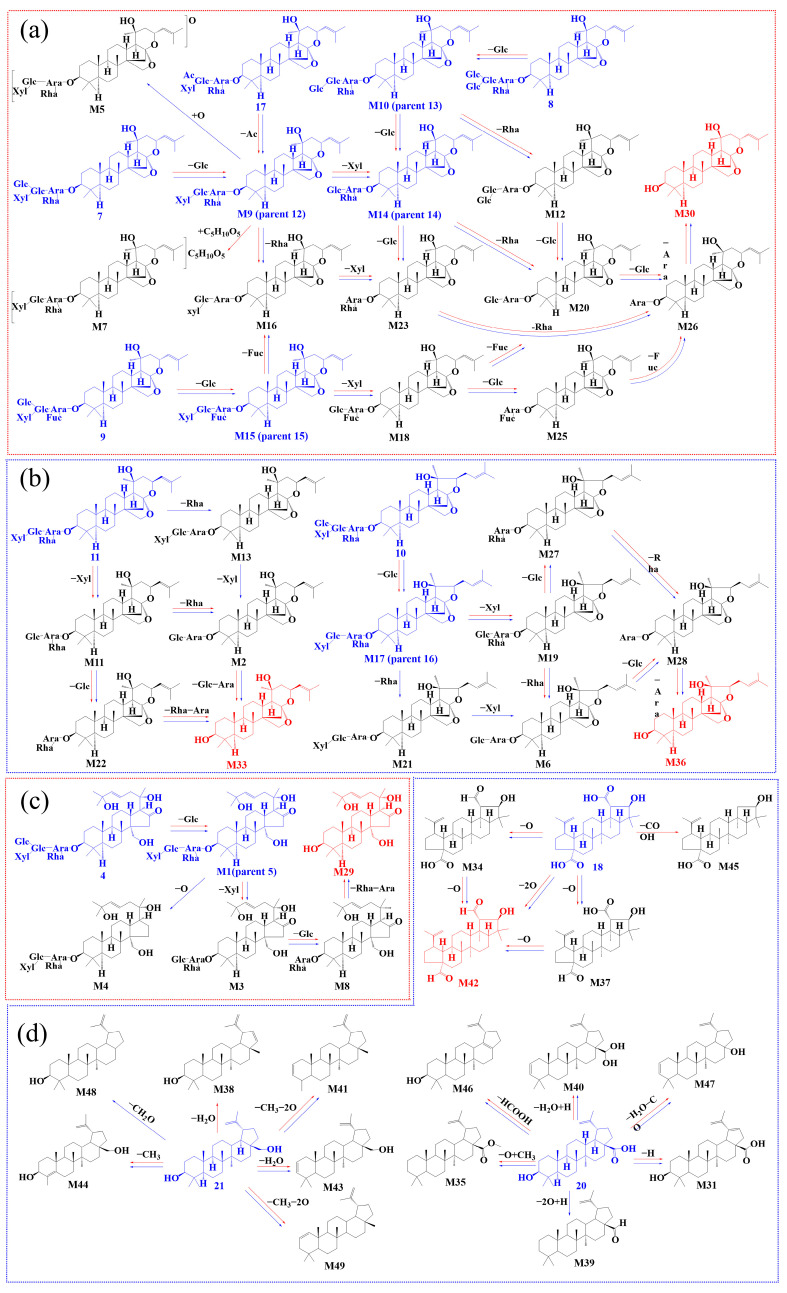
Elucidation of the metabolic pathways of ZSSS by intestinal microbiota from humans with insomnia and normal human: JS (**a**); C17-VJS (**b**); C16-KJS (**c**); PTSs (**d**). The red arrows indicate the metabolic processes mediated by normal intestinal bacteria, while the blue arrows indicate those mediated by insomnia-associated intestinal bacteria. The blue structures represent the original forms of ZSSS, whereas the red structures denote the ultimate metabolites of ZSSS.

As illustrated in Figure 2a, it is hypothesized that jujubogenin (M30) can be formed via three distinct pathways originating from the prototype compounds JuA (7), JuC (8), and JuA_1_ (9) through deglycosylation reactions. Notably, M23 serves as a common intermediate in all three pathways.

As the M7 generates at *m*/*z* 1221.5909 [M + HCOO]^−^, presumed to be C_57_H_92_O_25_, and produces fragmented ions at *m*/*z* 1175.5766, 1043.5440, 911.5012, 893.5158, 765.4428, 749.4489, 603.3911, and 47.3512, these fragments are associated with date kernels. The cleavage fragments of JuB (12) were completely consistent with the fragmentation pattern of M7. At the same time, M7 was calculated to have a higher molecular weight by approximately 132 Da compared with JuB (12). Hence, M7 was speculated to be a glycosylated product (JuB + C_5_H_10_O_5_ or C_5_H_8_O_4_), representing a unique reaction specific to group (3).

For C17-VJS, M11 exhibited an adduct molecular ion peak of [M + HCOO]^−^ at *m*/*z* 957.5053, consistent with the molecular formula C_47_H_76_O_17_. The presence of abundant fragment ions at *m*/*z* 911.5021, 749.4482, and 603.3865 supports the identification of M11 as a -Xyl metabolite of JuII (11).

C16-KJS compounds and their metabolites have been identified. Specifically, M3 is detected at *m*/*z* 975.5170 [M + HCOO]^−^. The molecular ion peak with the formula C_47_H_78_O_18_ generates fragment ions at *m*/*z* 929.5103, 767.4685, 749.4476, 603.3887, and 471.3466. The analysis indicates that these fragments result from the neutral loss of units (46 Da, 162 Da, 18 Da, 132 Da, and 146 Da), confirming that the metabolite M3 is a -Xyl derivative originating from JuG (5).

In addition, Table S2 lists 13 pentacyclic triterpenoid metabolites. Notably, M43 displays an [M + H]^+^ ion at *m*/*z* 425.3777, corresponding to the molecular formula C_30_H_48_O. Interestingly, the calculated molecular weight of M43 is 18 Da lower than that of BE (21), suggesting that M43 may be a dehydrated derivative of BE (21), while the metabolite may also be BE − H_2_O (M38).

However, compared with the N group, multiple oxidation and elimination metabolites, including compounds M4, M5, M13, M21, M39, and M48, were exclusively detected in the I group samples (Table S3). An M4 ion was observed at *m*/*z* 1047.5745 [M − H]^−^, corresponding to a molecular ion peak with the formula C_52_H_88_O_21_. Fragmentation analysis revealed ions at *m*/*z* 915.5337, 753.4813, and 607.4210, which are consistent with neutral losses of glycosyl fragments (132 Da, 162 Da, and 146 Da), indicating that metabolite M4 corresponds to JuG + 2H − O. For M5, mass spectrometry identified a molecular ion adduct peak at *m*/*z* 1105.5425, corresponding to the [M + HCOO]^−^ ion, suggesting the molecular formula C_52_H_84_O_22_. Further computational analysis confirmed additional fragments at *m*/*z* 1059.5427, 927.4971, 765.4439, and 619.3872, resulting from neutral losses of glycosyl fragments (132 Da, 162 Da, and 146 Da). These findings were corroborated by MS/MS spectra showing similar cleavage patterns for JuB, indicating that M5 is likely a product of JuB+O. Compounds M39 and M48, derived from pentacyclic triterpenoid components BA and BE, were identified as BA + H − 2O and BE − CH_2_O, respectively, based on their molecular weights of 426.3856 [M + H]^+^ and 413.2677 [M + H]^+^, along with the analysis of fragment losses.

In summary, the results demonstrate that the biotransformation of ZSSS in the intestinal tract is a complex process. Deglycosylation reactions involving stepwise cleavage of the sugar moieties are the main metabolic processes for TTSs, especially for the 26 common metabolites found in both the I group and N group samples. Meanwhile, the results show that the common metabolic reaction types of PTSs in ZSSS included elimination reactions, such as dehydrogenation, deoxygenation, dehydration, demethylation, and decarboxylation (Table S3).

### 2.3. Biotransformation Pathway

To further elucidate ZSSS metabolism, the biotransformation pathways mediated by gut microbiota were investigated in the metabolic profiles. Figure 2a illustrates the proposed metabolic pathways of JS through the gut microbiota of individuals with insomnia compared with those who are normal. The compound JuA (7) undergoes sequential deglycosylation to produce intermediates JuB (M9, 12), Ziz II (M14, 14), M23, and M26, ultimately yielding jujubogenin (M30). Additionally, M20 and M16 are generated through the sequential deglycosylation pathway. In addition, the removal of -Glc, -Xyl, -Glc, -Fuc, and -Ara groups from JuA_1_ (9) results in the formation of intermediates JuB_1_ (M15, 15), M18, M25, and M26, ultimately leading to the production of jujubogenin (M30). Moreover, different sequences of deglycosylation events also contribute to the formation of M16 and M20. Notably, among the aforementioned metabolites, M16, M20, and M26 serve as shared intermediates in both central pathways. In the last metabolic pathway, JuC (8) sequentially eliminates a Glc and a Rha to generate M12, or eliminates two Glc step by step to generate Ziz II (M14, 14). Subsequently, M23 and M26 are formed through deglycosylation reactions, ultimately leading to the synthesis of jujubogenin (M30). Notably, M12 is a unique metabolite in the pathway of JuC (8). The results indicated that deglycosylation is the predominant reaction for the transformation of JS into jujubogenin. The differences in JS metabolites between the gut microbiota of patients with insomnia and normal individuals are shown in Table S2. Notably, in the main biotransformation pathways, the peak areas of jujuboside metabolites containing 2–3 glucosyl units were significantly higher in the I group compared with the N group, as exemplified by Ziz II (M14, 14), JuB_1_ − Xyl (M18), and JuB_1_ − Xyl − Glc (M25), as shown in Table S6. The elevated levels of these metabolites in insomnia samples suggest a possible deficiency in the gut microbiota’s capacity to efficiently process glucosyl-containing metabolites with 2–3 glucosyl units, which may be attributed to a substantial decrease in gut microbiota biodiversity among patients with insomnia [14].

The metabolic pathways of C17-VJS via the gut microbiota of humans with insomnia and normal humans are illustrated in Figure 2b. Both JuII (11) and JuIV (10) undergo glycosyl hydrolysis reactions, ultimately generating their corresponding saponins M33 and M36. Similar metabolic pathways were observed in the gut microbiota transformation group. Notably, metabolites M13 and M21 were exclusively detected in the insomnia gut microbiota transformations, resulting from the removal of a rhamnosyl moiety. This indicates a higher diversity of glycosidases in the insomnia gut microbiota compared with the normal gut microbiota. Unfortunately, no intermediate metabolites of jujuboside E (JuE, 6) were detected in this study; only the final metabolite JuE − Glc − Rha − Xyl − Glc − Ara − Glc (M32) was identified. This may be due to the low initial content of JuE (6), leading to trace amounts of metabolites that fell below the detection limit. The relative percentages of the final metabolites M33 and M36 were lower at 6 h in the normal gut microbiota compared with the insomnia gut microbiota, but this trend reversed after 48 h.

Glycosyl hydrolysis and deoxygenation reactions were the primary metabolic process of C16-KJS (Figure 2c). Gut microbiota possess various deconjugating enzymes, such as *β*-D-glucosidases, which facilitate the deglycosylation of glycoside compounds [15]. JuH (4) can be sequentially deglycosylated by removing -Glc, -Xyl, -Glc, -Rha, and -Ara moieties to generate JuG (M1, 5), M3, M8, and M29. However, the deoxygenation product M4 was only detected in the transformed samples of gut microbiota from humans with insomnia. The removal of the -Glc moiety from protojujuboside A (PJuA, 1) generates PJuB (2), which undergoes a stepwise deglycosylation process, ultimately yielding protojujubogenin (M24). It is hypothesized that specific strains within the insomnia-associated human gut microbiota may participate in deoxygenation reactions, suggesting that deoxygenation is also a potential metabolic pathway for saponins in the gut microbiota [16].

For PTSs, deoxygenation, dehydration, decarboxylation, and demethylation were the predominant metabolic process in the gut microbiota samples from both normal humans and humans with insomnia (Figure 2d). The three prototype components CA (18), BA (20), and BE (21) all underwent dehydration, dehydrogenation, demethylation, and deoxygenation reactions. Specifically, the sequential loss of one oxygen atom from CA (18) resulted in the following two metabolites with distinct configurations: M37 and M42. Additionally, CA (18) was bioconverted to produce M45 via a decarboxylation reaction exclusively in the normal human gut microbiota. Meanwhile, BE (21) generated two metabolites with different configurations, M38 and M43, through a dehydration reaction. Notably, M38 was only detected in the normal human gut microbiota. BA (20) was bioconverted into M40 via a dehydration reaction and further lost both oxygen atoms to yield M39, which was exclusively observed in the gut microbiota of individuals with insomnia. Compared with TTSs, PTSs exhibited a higher degree of diversity in chemical reaction types.

In summary, the in vitro biotransformation of ZSSS extract by gut microbiota from normal humans and patients with insomnia revealed that the metabolic processes were complex. Deglycosylation was identified as the primary metabolic pathway for TTSs, whereas oxidation and elimination predominated for PTSs. Both the gut microbiota from normal humans and patients with insomnia primarily underwent deglycosylation, deoxygenation, dehydration, and deacylation reactions. However, unique metabolic pathways were also observed; glycosylation was exclusively detected in normal gut microbiota, while oxidation and hydrogenation were uniquely observed in the gut microbiota of patients with insomnia (Table S3).

### 2.4. Effect of ZSSS on the Microbial Communities

To explore the role of ZSSS on human microbiota communities after fermentation, 16S rRNA sequencing was used to analyze the composition of gut microbiota. The sequencing depth was sufficient for diversity measurement as shown by the rarefaction curve (Figure S5a). Additionally, the rank abundance curve exhibited a smooth and wide distribution (Figure S5b). The steady trends in the rarefaction curve and the rank abundance curve suggest that the sample size was adequate to reflect the species composition of the community, and the results derived from the data were valid.

Figure S5c shows that the Chao1 index of the I-0 h group was lower than that in the N-0 h group (*p* < 0.05), indicating that patients with insomnia have low microbial community diversity. Compared with the I-0 h group, both the Chao1 and observed species indices of the I-48 h group were significantly higher (*p* < 0.001) (Figure S5c,d). Furthermore, after ZSSS intervention, both the Shannon and Simpson indices suggested that the diversity of the fecal microbiota in the I-48 h group was significantly higher than in the I-0 h group (*p* < 0.01) (Figure S5e,f). These results indicates that the ZSSS intervention positively altered the diversity of the gut microbiota.

**Figure 3:**
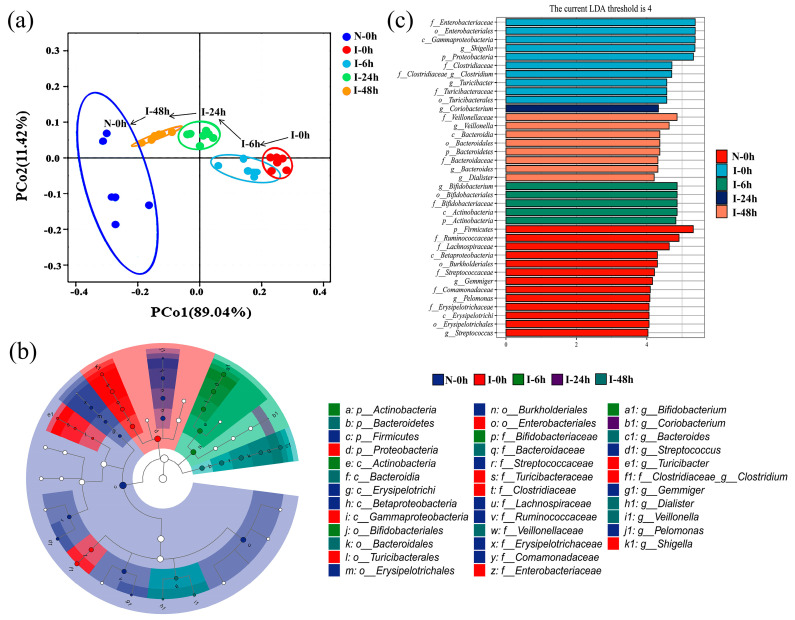
Gut microbiota analysis: PCoA score plots (**a**); linear discriminant analysis effect size (LEfSe) analysis (**b**); linear discriminant analysis (LDA) scores of each discriminant bacterial taxon and difference in dominant microorganisms under various treatments (**c**), and only the taxa with an LDA score higher than 4 are shown.

Based on the principal coordinate analysis (PCoA) diagram, the microbial community compositions from different groups exhibited obvious clustering (Figure 3a). The PCoA clearly demonstrates differences in the bacterial composition between the N-0 h and I-0 h groups, with a profound difference along the PCo1 axis (accounting for 89.04% of the overall variation). Interestingly, a time-dependent change was observed during fermentation. The dissimilarity of the gut microbiota community between the I-0 h group and the N-0 h group gradually diminished at fermentation durations of 6, 24, and 48 h. More strikingly, the I-48 h group was entirely located in the top left corner and partially overlapped with the N-0 h group, indicating that the two groups had a relatively similar community structure.

**Figure 4:**
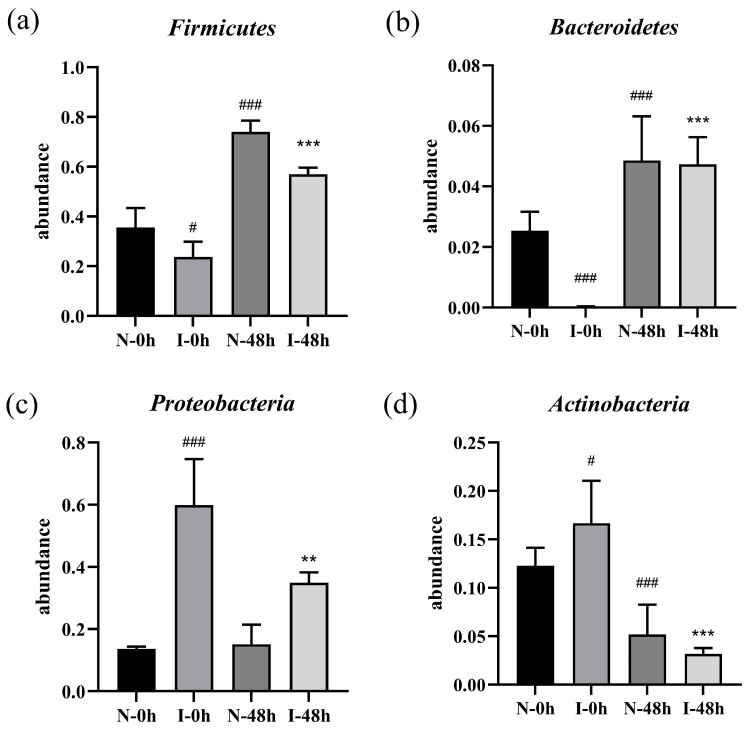
Relative abundances of the most abundant bacterial taxa at the phylum level during fermentation with ZSSS. *Firmicutes* (**a**); *Bacteroidetes* (**b**); *Proteobacteria* (**c**); *Actinobacteria* (**d**). ** *p* < 0.01 and *** *p* < 0.001 vs. theI-0h group. # *p* < 0.05 and ### *p* < 0.001 vs. the N-0h group (*n* = 6).

In addition, the species composition analysis at the phylum level showed a significant decrease in the abundance of *Firmicutes* (*p* < 0.05) and *Bacteroidetes* (*p* < 0.001), while there was a significant increase in the abundance of *Proteobacteria* (*p* < 0.001) and *Actinobacteria* (*p* < 0.05) in the I-0 h group compared with the N-0 h group. However, the ZSSS treatments (I-48 h) dramatically increased the abundance of *Firmicutes* (*p* < 0.001) and *Bacteroidetes* (*p* < 0.001), as well as significantly reduced the abundance of the *Proteobacteria* (*p* < 0.01) and *Actinobacteria* (*p* < 0.001), compared with the I-0 h group. These results are shown in Figure 4. Subsequently, the data on the top 10 abundant microbial populations at the genus level were analyzed, revealing an increase in the abundance of *Shigella* in the I-0 h group compared with the N-0 h group (Figure S6). The I-48 h group showed a notable reduction in the relative abundance of *Shigella* and an increase in the abundance of *Bifidobacterium, Bacteroides, Dorea, Dialister*, and *Sutterella* compared with the I-0 h group. Moreover, compared with the N-0 h group, the ZSSS-treated group (N-48 h) showed an increase in probiotics (e.g., *Bacteroides*, *Dialister*, and *Dorea*).

An LEfSe analysis was applied to determine the relative abundances of microbial taxa in the N-0 h, I-0 h, I-6 h, I-24 h, and I-48 h groups (Figure 3b,c). As a result, 37 differential genes were identified in the five groups of samples. *Gemmiger*, *Pelomonas*, and *Streptococcus* were found to be the dominant bacteria in the N-0 h group, meaning that these microbiota are present in normal humans. *Streptococcus* populations play a prominent role in the carbohydrate metabolism occurring in the small intestinal ecosystem [17].

The identification of *Turicibacter*, *Shigella*, and *Clostridium* as major bacteria in the I-0h group in this work is consistent with the findings of a study on circadian rhythm disturbance in mice with an abundance of *Turicibacter* in the colon microbiome [18]. Research has shown that disruptions to circadian rhythms may lead to serious negative effects, such as insomnia and cognitive impairment [19,20]. It is now widely known that peripheral insults causing a systemic inflammatory response can affect ongoing inflammation in the central nervous system (CNS), primarily through microglial activation and the production of inflammatory molecules, thus potentially exacerbating insomnia [21]. *Shigella* led to colonic inflammation, mucosal ulceration, and loss of intestinal barrier function [22]. Disruption of the intestinal barrier can result in the translocation of numerous microorganisms or microbial components into the systemic circulation, triggering an inflammatory response that subsequently impacts the CNS and exacerbates insomnia.

Nevertheless, the intake of ZSSS inhibits the growth of potential pathogens. *Bifidobacterium* was the dominant bacterial group in the I-6 h group. *Bifidobacterium* is a relatively common probiotic that secretes GABA in the gut, which can help improve sleep quality and duration [23]. With the increase in time, *Coriobacterium* became the prevailing flora in the I-24 h group. In the I-48 h group, *Veillonella*, *Dialister*, and *Bacteroides* were the predominant microbiota. Studies have found that depression is associated with a decrease in the beneficial bacterium *Dialister* [24]. Clinical studies have shown a significant reduction in the number of *Bacteroides* in patients with insomnia, with a significant increase observed following drug treatment [25].

In our study, we observed a decrease in microbial diversity and an increase in pathogenic bacteria among patients with insomnia. ZSSS enhanced the growth of beneficial bacteria and inhibited the proliferation of pathogenic bacteria, suggesting that it may improve sleep quality by restructuring the gut microbiota and increasing the abundance of beneficial bacteria.

### 2.5. SCFAs Production During Fermentation In Vitro

SCFAs, produced by beneficial microbiota in the microbiome, are believed to have many important roles in maintaining body health, such as protecting the intestinal mucosal barrier, reducing the level of inflammation, and enhancing gastrointestinal motility [26]. The retention time and characteristic ions of seven SCFA compounds are shown in Table S4. In addition, a standard curve was constructed with different concentrations of a standard mix containing acetic acid, propionic acid, isobutyric acid, butyric acid, isovaleric acid, valeric acid, and isocaproic acid, with 2-ethylbutanoic acid as an internal standard. Furthermore, as shown in Table S5, the inter-day precision, intra-day precision, stability, and repeatability were evaluated. As shown in Figure S7, seven kinds of SCFAs were effectively separated by GC-MS and were detected in the ZSSS fermentation samples. The SCFA contents at 0, 6, 24, and 48 h were determined by a Tukey test, and the results are shown in Table 1.

In the present study, we found a significant decrease in the concentration of SCFAs in the fecal content of patients with insomnia compared with normal humans. The total SCFAs content in the CN-48 h group was 13.10 ± 1.64 mmol/L, which was 2.23-fold higher than that observed in the CI-48 h group, possibly due to the lower concentration and weaker activity of the beneficial microbiota in patients with insomnia. As reported in the literature, saponins can increase SCFAs’ concentrations and maintain the balance of the gut microbiota [27]. Moreover, the production of total SCFAs increased from 2.79 ± 0.06 mmol/L (0 h) to 11.92 ± 0.43 mmol/L (48 h) in the I group, illustrating that ZSSS promote SCFAs production. Compared with the CN-48 h group, the contents of acetic acid, propionic acid, and butyric acid increased by 0.94-fold (20.43 ± 1.15 mmol/L), 1.04-fold (1.98 ± 0.10 mmol/L), and 4.16-fold (2.79 ± 0.26 mmol/L) in the N-48 h group, respectively. Similarly, compared with the CI-48 h group, the contents of acetic acid, propionic acid, and butyric acid increased by 0.91-fold (10.68 ± 1.56 mmol/L), 2.18-fold (0.35 ± 0.01 mmol/L), and 9-fold (0.10 ± 0.04 mmol/L) in the I-48 h group, respectively. The results of the SCFAs showed that ZSSS specifically enriched the contents of acetic acid, propionic acid, and butyric acid during the fermentation in the N group and I group.

As the main metabolites during the fermentation of carbohydrates and proteins, the production of SCFAs was associated with the abundance and structure of gut microbiota. Gut microbiota and its metabolites can affect the circadian rhythm of the host, while disruptions of circadian rhythms may lead to sleep disturbances in turn. As signaling molecules, SCFAs, particularly acetic acid and butyric acid, induced indirect modulation of circadian clocks in vivo. Acetic acid or butyric acid can also modulate the expression of the rhythm genes Per2 and Bmal1 [28]. Butyric acid potentially exerted epigenetic control on circadian rhythms because of its ability to inhibit NAD^+^-dependent histone deacetylases such as sirtuin-1 (SIRT1) [29]. Further, SCFAs exhibited effects on neural functions, such as enhancing sleep, modulating ghrelin receptor signaling, and contributing to the regulation of circadian rhythm [21]. Consequently, these findings suggested that ZSSS may have the potential to maintain circadian rhythms and improve host sleep by increasing SCFAs production.

### 2.6. Correlation Analysis of Gut Microflora and SCFAs

**Figure 5:**
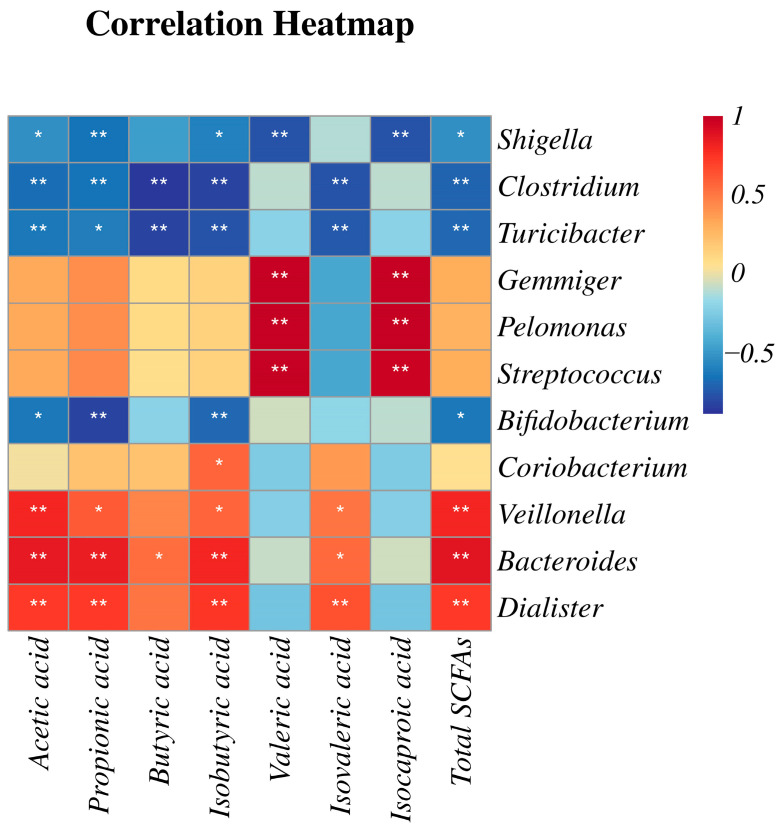
Pearson correlation coefficient between 7 SCFAs and 11 differential bacteria (* *p* < 0.05 and ** *p* < 0.01).

To comprehensively analyze the relationships between the gut microbiota and SCFAs, a correlation matrix was established by calculating the Pearson correlation coefficient, as shown in Figure 5. It revealed that *Gemmiger, Pelomonas*, and *Streptococcus* were significantly enriched in the N-0 h group, which demonstrated a significant positive correlation with the syntheses of valeric acid and isocaproic acid. In contrast, *Shigella*, *Clostridium*, and *Turicibacter* were notably enriched in the I-0 h group, which showed a negative correlation with almost all SCFAs. The studies demonstrate that SCFAs inhibited the colonization of *Shigella* to maintain a stable microbial environment [30], and propionic acid significantly downregulated the abundance of *Turicibacter* in the cecum [31].

Remarkably, the structure of the gut microbiota community was completely reshaped by the ZSSS intervention after 48 h. *Veillonella*, *Bacteroides*, and *Dialister* exhibited significant positive correlations with acetic acid, propionic acid, isobutyric acid, isovaleric acid, and total SCFAs, with slightly different significances. Acetic acid has previously been demonstrated to modulate the levels of neurotransmitters, such as glutamate, glutamine, and GABA_A_, in the hypothalamus while also promoting the expression of anorexigenic neuropeptides. Propionic acid was predominantly produced via the succinate pathway by certain *Firmicutes*, including *Veillonella* and *Dialister* [32]. Propionic acid inhibited histone deacetylase (HDAC) activity, modulated the inflammatory response, and, consequently, exerted various neurological effects, including enhancing sleep [21]. Notably, only *Bacteroides* exhibited a significant positive correlation with butyric acid production (*p* < 0.05). *Bacteroides* have been identified as key producers of butyric acid [33]. Studies suggest that increased butyric acid production may play a pivotal role in mediating the gut microbiota-related effects of melatonin on cognitive impairment caused by sleep deprivation. Additionally, butyric acid can significantly enhance non-rapid eye movement (NREM) sleep in rodents [34]. Accumulating evidence indicates that SCFAs produced by the gut microbiota serve as important sources of sleep-promoting signals, particularly propionic acid and butyric acid. Our findings suggest that ZSSS treatment may improve sleep quality by increasing the abundance of beneficial microbial species and promoting intestinal SCFAs production.

### 2.7. ZSSS Pharmacodynamics Analysis

**Figure 6:**
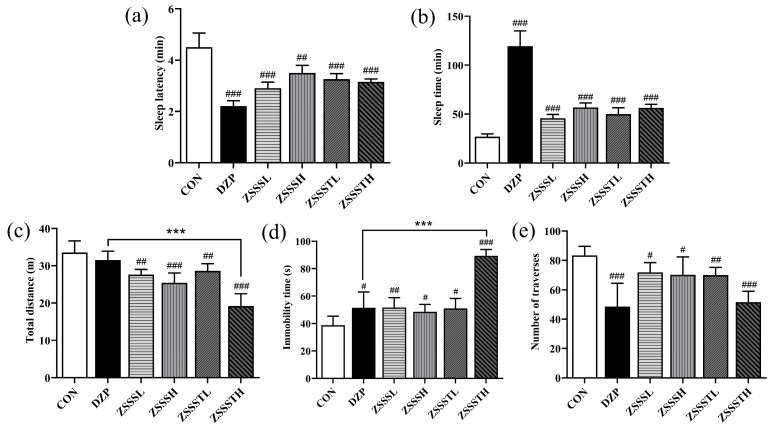
Effects of the administration of ZSSS and ZSSST on sleep latency (**a**) and sleep time (**b**) in mice induced with sodium pentobarbital (44 mg/kg, i.p.) (*n* = 6). Inhibitory effects of ZSSS and ZSSST on spontaneous locomotor activity in mice, total distance (**c**), immobility time (**d**), and number of traverses (**e**) (*n* = 6). # *p* < 0.05, ## *p* < 0.01, and ### *p* < 0.001, vs. the CON group, *** *p* < 0.001 vs. the DZP group.

In order to investigate the difference in sedative–hypnotic actions between ZSSS and ZSSST groups, SPST and OFBT, two classic behavioral pharmacology methods to evaluate sedative–hypnotic activity, were performed. As shown in Figure 6a,b, all drug groups significantly reduced the sleep latency of mice (*p* < 0.001) and significantly prolonged the sleep time of mice (*p* < 0.001) compared with the CON group. The results showed that ZSSS and ZSSST had a synergistic effect with pentobarbital to prolong sleep time in mice.

The sedative activities of ZSSS and ZSSST were investigated by recording the spontaneous locomotor activity of mice. Compared with the CON group, the total distance of the ZSSS group and ZSSST group decreased significantly (*p* < 0.001), the immobility time increased (*p* < 0.05), and the number of traverses penetrated decreased (*p* < 0.05) (Figure 6c–e). The findings demonstrate that both ZSSS and ZSSST reduced the spontaneous locomotor activity of mice. Notably, compared with the diazepam (DZP) group, the total distance traveled and immobility time of the ZSSSTH group showed significant differences (*p* < 0.001), indicating that ZSSSTH exhibited the most effective sedative effect. It is speculated that gut-microbiota-induced deglycosylation can decompose sugar moieties from ZSSS, thereby producing secondary glycosides and aglycones that are more easily absorbed in the intestines and exert higher bioavailability than their parent compounds.

### 2.8. Molecular Docking Studies

Molecular docking was used in our study to validate the interaction between the metabolites and related targets. GABA_A_ receptors modulated neurotransmission in the central nervous system and were regarded as target protein for treating insomnia. 5-HT was one of the most important neurotransmitters for sleep regulation [35]. The interaction of all metabolites with the GABA_A_ receptor (PDB ID: 6HUP) and 5-HT_2a_ receptor (PDB ID: 6A94) was studied by molecular docking analysis. Detailed information on the molecular docking is shown in Table S6. The binding energy of the ZSSS prototype compounds and their metabolites docking with 5-HT_2a_ receptor was generally greater than that with the GABA_A_ receptor. Generally, a lower binding energy indicates a more stable ligand–receptor binding conformation. The molecules that showed a strong interaction with both the GABA_A_ and 5-HT_2a_ receptors were limited in number, such as JuE − Glc − Rha − Xyl − Glc − Ara − Glc (M32, GABA_A_: −9.35 kcal/mol; 5-HT_2a_: −7.59 kcal/mol) and CA − COOH (M45, GABA_A_: −9.46 kcal/mol; 5-HT_2a_: −3.25 kcal/mol). This finding is consistent with our previous work, which showed that the primary target of ZSSS was the GABA_A_ receptor [36].

As described in Figure 2a, JuA (7) could be metabolized to jujubogenin through deglycosylation step by step. Interestingly, the deglycosylation products, such as JuB (12, −2.55 kcal/mol), JuII (11, −4.99 kcal/mol), JuB_1_ − Xyl − Glc − Fuc (M26, −8.17 kcal/mol), and jujubogenin (M30, −8.83 kcal/mol), have a lower binding energy with GABA_A_ than JuA (7, 1.61 kcal/mol). The binding energies of the prototype components, such as JuG (5, 0.42 kcal/mol), JuII (11, −4.99 kcal/mol), and BA (20, −7.78 kcal/mol), with the GABA_A_ receptor were found to be higher than those of the corresponding final metabolites, JuG − Xyl − Glc − Rha − Ara (M29, −6.4 kcal/mol), JuII − Xyl − Glc − Rha − Ara (M33, −9.8 kcal/mol), and BA − O + CH_3_ (M35, −8.67 kcal/mol), respectively.

**Figure 7:**
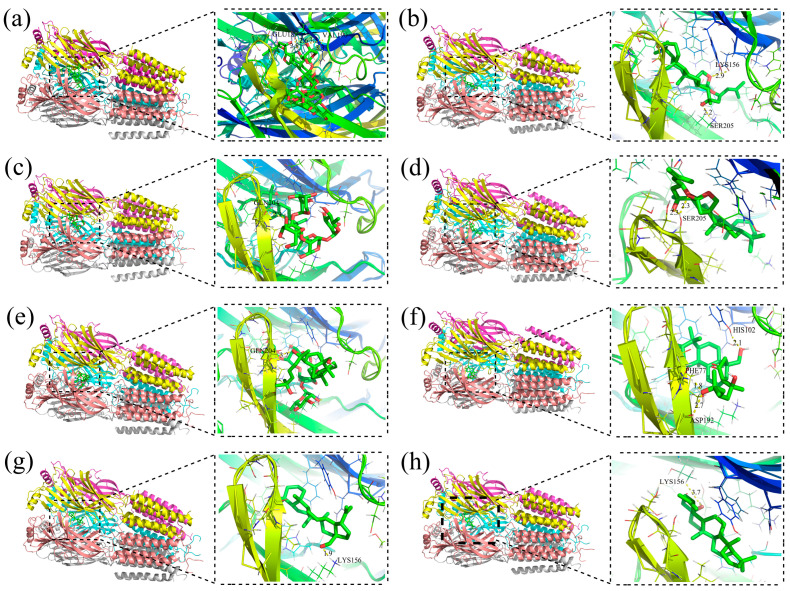
Docking of four representative prototype saponins of ZSSS and their final metabolites on the GABA_A_ receptor protein. Docking of JuA (**a**), jujubogenin (**b**), JuII (**c**), M33 (**d**), JuG (**e**), M29 (**f**), BA (**g**), and M35 (**h**) on the GABA_A_ receptor protein, respectively. The colorful part is the protein receptor, the green part is the small-molecule ligand, and the pink part is the hydrogen bond formed between the ligand and the receptor.

The four representative prototype saponins and their final corresponding metabolites were subjected to docking with GABA_A_. The 3D diagram illustrated the relevant docking pocket of the GABA_A_ receptor and saponin components, and their respective docking sites are shown in Figure 7. The prototype components usually formed one hydrogen bond with an amino acid; for example, JuG established a hydrogen bond contact with GLN204. The final metabolites formed multiple hydrogen bonds with amino acids, such as M29, which established three hydrogen bonds with ASP192, PHE77, and HIS102. Through the biotransformation of the gut microbiota, the resulting metabolites exhibited a higher binding energy and more compatible binding conformation at the GABA_A_ binding site.

## 3. Materials and Methods

### 3.1. Chemicals and Reagents

In the reference standards, JuA, CA, AA, BA, and BE were obtained from Baoji Herbest Co. (Baoji, China), and JuB_1_, PJuB, and JuB were purchased from Nanjing Spring & Autumn Biotech Co. (Nanjing, China). The SCFA standards, including acetic acid, propionic acid, isobutyric acid, butyric acid, isovaleric acid, valeric acid, isocaproic acid, and 2-ethylbutyric acid were purchased from Tokyo Chemical Industry (TCI) Development Co., Ltd. (Shanghai, China). The purity of each standard was greater than 98%. The general anaerobic medium (GAM) and Duchenne phosphate buffer (DPBS) were purchased from Beijing Solaibao Technology Co., Ltd. (Beijing, China). The HPLC-MS-grade acetonitrile (ACN), methanol, and formic acid (FA) were purchased from Fisher Scientific (Shanghai, China). Other reagents were of analytical grade.

The ZSS was purchased from Hebei Anguo Jiarun TCM Co. (Anguo, China), and authenticated by Professor Xiangping Pei from Shanxi University of Chinese Medicine. A voucher specimen (No. 20170901001) has been deposited at the Shanxi University of Chinese Medicine.

### 3.2. Preparation of ZSSS

ZSS (4 kg) was pulverized into a powder and then extracted by refluxing with petroleum ether for two cycles (*v*/*v*, 1:10, 1 h per cycle). Then, the residues were extracted by heat-refluxing with 70% ethanol twice (*v*/*v*, 1:10, 2 h per extraction). All filtrates were concentrated to 1 g/mL (crude drug per mL). The filtrates were loaded onto AB-8 macroporous resin and sequentially eluted with distilled water, 10%, 30%, 50%, and 70% ethanol. Finally, the 70% ethanol fraction was collected and freeze-dried to obtain ZSSS. The total saponins content of the ZSS was determined by ultraviolet (UV) analysis, and the purity of the ZSSS was 54.22%.

### 3.3. Fecal Sample Collection and In Vitro Fermentation of ZSSS

The study’s protocol for the fecal sample collection from normal volunteers and patients with insomnia was approved by the local ethics committee of Shanxi University of Chinese Medicine. Normal fecal samples were obtained from six healthy volunteers (three males and three females). Fecal samples from six patients with insomnia (three males and three females) were collected from the Affiliated Hospital of Shanxi University of Chinese medicine (Shanxi, China). None of the volunteers had taken any antibiotics for at least six months before this study. Information on the healthy volunteers and the patients with insomnia is provided in Table S7.

A total of five groups were included in the study, as follows: (1) incubations of ZSSS in GAM lacking intestinal microbiota; (2) incubations of the intestinal microbiota from normal humans in GAM without ZSSS (CN group); (3) incubations of the intestinal microbiota from normal humans in GAM with ZSSS (N group); (4) incubations of the intestinal microbiota from patients with insomnia in GAM without ZSSS (CI group); and (5) incubations of the intestinal microbiota from patients with insomnia in GAM with ZSSS (I group). The preparation of the fecal suspension was as follows: equal amounts of feces were moved into a sterile vial and immediately homogenized with sterilized DPBS to generate a 10% (*v*/*v*) suspension. Then, the slurry was filtered through two layers of sterile gauze sponges and centrifuged at 2000 rpm (4 °C) for 10 min. The GAM was prepared according to our previous method with some modifications [37]. The fecal suspension at 3 mL was mixed with 30 mL of pre-sterilized GAM, and then incubated at 37 °C in an anaerobic incubator for 9 h. An aliquot of 1.0 mL each of sterile ZSSS (10 mg/mL) was added to 10 mL of human fecal suspension, and the mixture was blended and incubated 37 °C. The samples were collected at 0, 6, 12, 24, and 48 h, respectively, with 1 mL collected each time and frozen and stored at −80 °C for further study.

### 3.4. Sample Preparation

All samples from the five groups at each time point were individually purified using a Tri-Pak SPE C18 column (solid-phase extraction) and sequentially eluted with pure water, 40% methanol, and 100% methanol. The 100% methanol fraction was collected and evaporated to dryness. The residue was then dissolved in 50% acetonitrile (2 mL) and filtered through a 0.22 μm membrane. The resulting supernatant was analyzed by UPLC-Q-Orbitrap-MS.

### 3.5. UHPLC-Q-Orbitrap-MS Analysis

The optimum chromatographic separation was achieved on a Dionex UltiMate 3000 UPLC system coupled with a Q-Orbitrap-MS (Thermo Scientifc, Bremen, Germany) using an Agilent Proshell 120 EC-C_18_ (3.0 × 100 mm, 2.7 μm) column at a flow rate of 0.3 mL/min at 30 °C. The mobile phase consisted of 0.1% FA water (*v*/*v*) (A) and ACN (B). A gradient program was established as follows: 0–5 min, 30–35% B, 5–10 min, 35–45% B, 10–15 min, 45–65% B, 15–20 min, and 65–100% B. The column was equilibrated for 5 min prior to each analysis. The sample injection volume was 1.0 µL.

For MS analysis, the Q-Orbitrap-MS was combined with heat electrospray ionization (ESI) source and operated in data-dependent MS scan (dd-MS^2^) mode through negative and positive switching modes with the following parameter settings: spray voltage of +3.2 KV and −2.5 KV; sheath gas of 35 for ESI (±); probe heater temperature of 350 °C for ESI (±); auxiliary gas pressure of 10 for ESI (±); and NCE (normalized collision energy) settings at 30%, 40%, and 50% in the negative ion mode and 50% in the positive ion mode. The full-scan mode range was set at 50–1500 *m*/*z*, and the resolution was set to 70000. Data were processed using the Xcalibur^TM^ 3.0.63 software (Thermo Fisher Scientific Inc., Waltham, MA, USA).

### 3.6. 16S rRNA Sequencing Analysis

The fecal genomic DNA was extracted with the QiAamp DNA stool Mini Kit (Qiagen, Hilden, Germany). Prior to sequencing, the 16S rRNA V3–V4 regions from each sample were amplified using primers targeting these regions. Sequencing of the bacterial 16S rRNA V4 region was performed on an Illumina MiSeq platform by Piseno Biotechnology Co., Ltd. (Shanghai, China), using a commercial standard pipeline. Raw sequencing reads were processed by the DADA2 package (version 1.8) based on a 97% similarity threshold. A principal coordinate analysis (PCoA) and hierarchical cluster analysis were performed using the Bray–Curtis distance matrix and the average method according to the abundances of the OTUs using QIIME (version 1.7.0) and R software (version 2.15.3). The statistical significance of the separation among the groups was evaluated by the linear discriminant analysis effect size (LEfSe) method based on the linear discriminant analysis (LDA) scores.

### 3.7. Determination of the SCFAs in Fecal Samples

The SCFA concentration was measured according to the previous method with some modifications [38]. A volume of 1 mL of 50% (*w*/*v*) methanol was added to 1 mL of the fermentation samples, and then 60 μL of 0.09 M H_2_SO_4_ solution was added to adjust the pH to 2–3. 2-Ethylbutanoic acid, as an internal standard, was added. The supernatants were then obtained by centrifugation (8000 rpm, 10 min, and 4 °C), filtered through 0.22 μm filters, and stored at −20 °C until analysis. The SCFAs in the samples were analyzed using an Agilent 7890 B gas chromatography instrument coupled with an Agilent 5977 B Mass Selective Detector (Agilent Technologies, Santa Clara, CA, USA).

### 3.8. Animals and Treatment

The ICR male mice (Grade SPF, Beijing Viton Lihua Experimental Animal Technology Co., Ltd., license no. SCXH (Jing) 2021-0011, Beijing, China), weighing 18 ± 2 g, were used. The mice were housed in cages at 24 °C, 55% controlled humidity, and 12 h light/dark cycle, limiting access to food and water. Mice were randomly divided into the following six groups: control (CON) group, diazepam (DZP, 2 mg/kg) group, low-dose ZSSS (ZSSSL, 0.18 g/kg) group, high-dose ZSSS (ZSSSH, 0.36 g/kg) group, low-dose ZSSS transformation product (ZSSSTL, 0.14 g/kg) group, and high-dose ZSSS transformation product (ZSSSTH, 0.28 g/kg) group. Detailed information is provided in Table S8. Six groups of mice were administered treatments for 7 consecutive days. After the 6 groups of mice were treated continuously for 7 days, pharmacodynamic experiments were carried out.

### 3.9. Behavioral Test

#### 3.9.1. Open-Field Behavior Test

On the fourth day of administration, 30 min after dosing, the mice were placed in the central area of the open-field apparatus (XR-XZ 301, Shanghai Xinsoft Information Technology Co., Ltd., Shanghai, China). After allowing the mice to adapt for 2 min, the total distance traveled, resting time, and the number of grids crossed by each mouse were recorded over a 5 min observation period.

#### 3.9.2. Evaluation of Sleep Onset and Sleep Duration

In the present study, 44 mg/kg pentobarbital (intraperitoneal injection, i.p.) was used as the hypnotic dose. Mice were placed on their backs and observed for the onset of sleep following the pentobarbital injection. The criterion for a mouse to fall asleep was that the mouse lost the righting reflex and remained on its back for more than 1 min. The sleep latency (the time between pentobarbital administration and the disappearance of the righting reflex) and the sleep duration (the time between the righting reflex disappearing and waking up) were recorded for each mouse. Mice that did not fall asleep within 30 min were excluded.

### 3.10. Molecular Docking

In order to further verify the potential interactions of the metabolites with neurotransmitter receptors (GABA_A_ and 5-hydroxytryptamine receptor 2A, 5-HT_2a_), molecular docking simulation studies were performed using AutoDock 4.2 with the Lamarckian Genetic Algorithm (LGA) as described by Yu et al. [39]. The 3D structure of the GABA_A_ (PDB ID: 6HUP) and 5-HT_2a_ (PDB ID: 6A94) were retrieved from the RCSB Protein Data Bank (http://www.rcsb.org, accessed on 14 January 2021) [40,41]. Before molecular docking, the ligand was prepared by optimizing structures and minimizing energy under the MM2 force field with ChemBio Office 2019. A cubic grid box of 60 Å in size (x, y, and z) with a spacing of 0.375 Å was generated along with grid maps. Prior to the docking screening experiments, the co-crystallized ligand was removed, and the prepared ligands were docked to the protein using identical parameters. Based on the docking results, the best-scoring docking model with the lowest docking energy was selected to represent the optimal ligand-binding site of the compound. For each ligand, the interaction energy was calculated to define the interactions between the ligand and the receptor.

### 3.11. Statistical Analysis

All experimental data are presented as the mean ± standard deviation (SD). An unpaired two-tailed *t*-test was used to assess the differences in the SCFAs’ concentrations. A data analysis involving more than two groups was performed with one-way analysis of variance (ANOVA), least significant difference (LSD) test, and Duncan’s multiple range test via SPSS software (IBM SPSS Statistics Ver. 23). A *p*-value < 0.05 was considered statistically significant in this study.

## 4. Conclusions

In this study, we investigated the in vitro biotransformation of Ziziphi Spinosae Semen saponins by gut microbiota from healthy and insomniac groups. The results show that the metabolic profiles of ZSSS, as influenced by the gut microbiota in patients with insomnia, were significantly distinct from those of healthy people. Notably, the abundances of metabolites containing 2–3 glucosyl residues were elevated, and unique oxidation and hydrogenation were observed. These findings may be associated with the enhanced activity of specific enzymes within the gut microbiota of patients with insomnia.

After the ZSSS intervention, the gut microbiota structure exhibited significant changes, characterized by the proliferation of probiotics (*Veillonella*, *Dialister*, and *Bacteroides*) and the suppression of potential pathogenic bacteria (*Shigella* and *Turicibacter*). Additionally, ZSSS enhanced the synthesis of SCFAs in the gut microbiota, particularly acetic acid, propionic acid, and butyric acid. SCFAs are not merely metabolites produced by the gut microbiota but also serve as critical mediators linking the gut microbiota and brain function. Notably, *Bacteroides* demonstrated a strong positive correlation with the synthesis of these three SCFAs. Several studies have confirmed that SCFAs, including acetic acid, propionic acid, and butyric acid, can penetrate the blood–brain barrier and influence the synthesis and release of 5-HT and its receptors, thereby modulating sleep [42]. Furthermore, SCFAs can improve sleep quality by attenuating excessive activation of the hypothalamic–pituitary–adrenal (HPA) axis and regulating associated inflammatory factors [43]. In this study, ZSSS significantly increased SCFA levels, potentially enhancing brain–gut axis function and exerting anti-inflammatory effects to improve sleep.

In addition, the ZSSST exhibited the strongest sedative and hypnotic effects in both SPST and OFBT, significantly reducing voluntary activities in mice. The MDE analysis revealed that metabolites with fewer glycosidic bonds demonstrated a stronger binding affinity to the GABA_A_ receptors. This suggests that the active components of ZSSS may exert their effects on the central neurotransmitter system via secondary glycosides or aglycones, which are more readily absorbed by the intestine and exhibit higher bioavailability compared with the prototype compounds. This study systematically investigated the differences in the in vitro biotransformation of ZSSS by the gut microbiota of healthy and insomniac populations, uncovering a novel mechanism for ZSSS-induced sleep improvement. These findings provide valuable insight into the efficacy and mechanism of action of ZSSS, paving the way for future research.

## Figures and Tables

**Table 1:** The contents of seven SCFAs by GC-MS.

Treatment	Time (h)	SCFAs (mmol/L)							
		Acetic Acid	Propionic Acid	Isobutyric Acid	Butyric Acid	Isovaleric Acid	Valeric Acid	Isocaproic Acid	Total SCFAs
N	0	7.52 ± 0.79 ^fg^	0.30 ± 0.05 ^c^	0.10 ± 0.01 ^abcd^	0.09 ± 0.01 ^ab^	0.14 ± 0.02 ^a^	0.14 ± 0.01 ^abc^	0.01 ± 0.01 ^a^	8.29 ± 0.82 ^fg^
	6	8.01 ± 0.14 ^fg^	0.96 ± 0.12 ^e^	0.14 ± 0.03 ^bcd^	0.89 ± 0.04 ^e^	0.17 ± 0.03 ^a^	0.15 ± 0.06 ^bcd^	0.01 ± 0.01 ^a^	10.33 ± 0.29 ^gh^
	24	13.63 ± 0.44 ^i^	1.69 ± 0.01 ^f^	0.22 ± 0.10 ^d^	1.70 ± 0.13 ^f^	0.59 ± 0.10 ^de^	0.18 ± 0.06 ^cd^	0.04 ± 0.01 _b_	18.04 ± 0.39 ^j^
	48	20.43 ± 1.15 ^j^	1.98 ± 0.10 ^g^	0.60 ± 0.11 ^f^	2.79 ± 0.26 ^g^	0.85 ± 0.06 ^f^	0.21 ± 0.03 ^d^	0.06 ± 0.03 ^c^	26.87 ± 1.56 ^k^
CN	0	6.91 ± 0.79 ^ef^	0.22 ± 0.01 ^bc^	ND	0.22 ± 0.01 ^ab^	ND	ND	ND	7.34 ± 0.38 ^ef^
	6	6.84 ± 0.14 ^ef^	0.72 ± 0.05 ^d^	0.12 ± 0.01 ^abcd^	0.30 ± 0.01 ^bc^	0.12 ± 0.02 ^a^	0.08 ± 0.01 ^a^	ND	8.19 ± 0.44 ^f^
	24	8.85 ± 0.46 ^gh^	0.94 ± 0.07 ^e^	0.37 ± 0.04 ^e^	0.52 ± 0.01 ^cd^	0.22 ± 0.04 ^ab^	0.09 ± 0.01 ^ab^	ND	10.98 ± 0.46 ^h^
	48	10.54 ± 1.15 ^h^	0.97 ± 0.14 ^e^	0.50 ± 0.03 ^ef^	0.54 ± 0.06 ^d^	0.43 ± 0.02 ^c^	0.12 ± 0.02 ^abc^	ND	13.10 ± 1.64 ^i^
I	0	2.30 ± 0.39 ^ab^	ND	0.17 ± 0.05 ^cd^	0.01 ± 0.01 ^a^	0.30 ± 0.01 ^b^	ND	ND	2.79 ± 0.06 ^abc^
	6	3.70 ± 0.40 ^bc^	ND	0.05 ± 0.04 ^abc^	0.11 ± 0.05 ^ab^	0.57 ± 0.06 ^de^	ND	ND	4.43 ± 0.16 ^bcd^
	24	3.90 ± 0.31 ^bcd^	0.20 ± 0.04 ^bc^	0.11 ± 0.02 ^abcd^	0.10 ± 0.04 ^ab^	0.52 ± 0.02 ^cd^	ND	ND	4.83 ± 0.08 ^cd^
	48	10.68 ± 1.56 ^h^	0.35 ± 0.01 ^c^	0.14 ± 0.04 ^abcd^	0.10 ± 0.04 ^ab^	0.65 ± 0.01 ^e^	ND	ND	11.92 ± 0.43 ^hi^
CI	0	1.37 ± 0.02 ^a^	ND	ND	ND	ND	ND	ND	1.37 ± 0.05 ^a^
	6	2.67 ± 0.16 ^abc^	ND	ND	ND	ND	ND	ND	2.67 ± 0.65 ^ab^
	24	4.28 ± 0.06 ^cd^	ND	ND	ND	ND	ND	ND	4.28 ± 0.26 ^bcd^
	48	5.58 ± 0.50 ^de^	0.11 ± 0.01 ^ab^	0.03 ± 0.02 ^ab^	0.01 ± 0.01 ^ab^	ND	ND	ND	5.87 ± 0.62 ^de^

a–k: The mean value in the same column with different letters was significantly different (*p* < 0.05), as determined by a Tukey test; ND: undetected.

## Data Availability

The datasets developed and/or analyzed during the current study are available from the corresponding author upon reasonable request.

## Supplementary Materials

The following supporting information can be downloaded at: https://www.mdpi.com/article/10.3390/ijms26094011/s1.

## Abbreviations

ZSS: Ziziphi Spinosae Semen; ZSSS: Ziziphi Spinosae Semen saponins; SPST: sodium pentobarbital-induced sleeping test; OFBT: open-field behavior test; MDE: molecular docking experiment; TTSs: tetracyclic triterpenoids saponins; PTSs: pentacyclic triterpenoids saponins; GAM: general anaerobic medium; ESI: electrospray ionization; SCFAs: short-chain fatty acids; GABA_A_: γ-amino-butyric acid A; PCoA: principal coordinate analysis; LEfSe: linear discriminant analysis effect size; LDA: linear discriminant analysis; DZP: diazepam; ZSSSL: low-dose ZSSS; ZSSSH: high-dose ZSSS; ZSSSTL: low-dose ZSSS transformation product; ZSSSTH: high-dose ZSSS transformation product; LGA: Lamarckian genetic algorithm; SD: standard deviation; ANOVA: one-way analysis of variance; LSD: least significant difference; JS: jujuboside saponin; C16-KJS: C16-keto jujubogenin-type saponin; C17-VJS: C17-side-chain-varied jujubogenin-type saponin; CNS: central nervous system; HDAC: histone deacetylase; NREM: non-rapid eye movement.
