# Supplementary material for: In Vitro Biotransformation of Ziziphi Spinosae Semen Saponins by Gut Microbiota from Healthy and Insomniac Groups
Source: Int J Mol Sci. 2025 Apr 24;26(9):4011. doi: 10.3390/ijms26094011 (PMC12072027; doi:10.3390/ijms26094011)
Supplement: Supplementary file 1 [file ijms-26-04011-s001.zip › ijms-3526071-supplementary.pdf]

Supporting information

In Vitro Biotransformation of Ziziphi Spinosae Semen Saponin by Gut Microbiota from Healthy and Insomnia Groups

Xiaofang Cui [1,†], Shengmei Zhang [1,†], Ling He [1], Huizhu Duan [1], Yujun Xie [1], Xiangping Pei [1], Yan Yan [2,*] and Chenhui Du [1,*]

[1]School of Chinese Materia Medica, Shanxi University of Chinese Medicine, Taiyuan 030619, China; xiaofangcui2025@163.com (X.C.); 13145263888@163.com (S.Z.); 13994154651@163.com (L.H.); duanhz0506@163.com (H.D.); yujun_xie@163.com (Y.X.); peixp69@163.com (X.P.)

[2]Modern Research Center for Traditional Chinese Medicine, Shanxi University, Taiyuan 030006, China

*Correspondence: yanyan520@sxu.edu.cn (Y.Y.); dch@sxtcm.edu.cn (C.D.)

[†]These authors contributed equally to this work.

GC-MS analysis. An DB-FFAP (30 m × 0.25 mm × 0.25μm) capillary column (Agilent Technologies, USA) was used for the separation. Helium (≥ 99.999%, China) was used as the carrier gas at a flow rate of 1.0 mL/min. The initial column temperature was set at 90 °C, which was maintained for 1 min, then raised to 200 °C at 10 °C/min, and held on for 13 min. The temperature of ion source, injector and transfer line were set to 280 °C, 230 °C and 150 °C. During qualitative analysis, the mass spectral data were collected in a full-scan mode over a mass range 30-500 *m/z* with an electron energy of 70 eV, while selected ion monitor (SIM) mode was applied for quantitative analysis. The injection volume was 0.5 μL. The quantitative analysis was performed using Agilent Mass Hunter Quantitative Analysis (version B.08.00) software. A standard curve was generated by employing various concentrations of a standard mixture comprising acetic acid, propionic acid, isobutyric acid, butyric acid, isovaleric acid, valeric acid, and isocaproic acid alongside 2-ethylbutanoic acid as an internal reference compound. In addition, the inter-day precision, intra-day precision, stability and repeatability were investigated.

The preparation of ZSSST. The patient's feces were vortexed and suspended using 10 times the amount of pre-sterilized DPBS. After filtering through two layers of gauze, the filtrate was centrifuged at 2000 rpm for 10 min, and the supernatant was taken to obtain the fecal suspension containing human intestinal flora. Take 150 mL of the fecal suspension, add 10 times GAM, and anaerobically culture at 37 ºC for 9 h. Make three parallels. After 9 h, add 3 g ZSSS to each portion, and take it out after culturing for 24 h. Concentrate under reduced pressure to 400 mL. The filtrates were loaded onto AB-8 macroporous resin and sequentially eluted with distilled water, 10%, 30%, 50%, and 70% ethanol. Finally, the 70% ethanol fraction was collected and freeze-dried to obtain ZSSST, obtaining 7.03 g of ZSSST.

Figure S1. A diagram for rapid identification of triterpenoids by UPLC-Q-Orbitrap-MS.

Figure S2. The fragmentation pathways of JuA in the negative and postive MS/MS (**a**); the fragmentation pathways of JuII in the negative and postive MS/MS (**b**); the fragmentation pathways of PJuB in the negative MS/MS (**c**); the fragmentation pathways of glucose residue ion in the negative MS/MS (**d**). The blue parts represent characteristic ions.

Figure S3. Chemical structures of ZSSS compounds and their metabolites.

Figure S4. Total ion chromatograms (TIC) in positive mode, ZSSSS and GAM (**a**); CN group (**b**); N-48 h group (**c**); CI group (**d**); I-48 h group (**e**).

Figure S5. Rarefaction Curve (**a**); Rank abundance curve (**b**); Effects of ZSSS treatment on the α-diversity of fecal microbiota; Chao1 index (**c**); Observed species index (**d**); Shannon index (**e**); Simpson index of each group (n = 6) (**f**). Values are presented as the mean ± SEM. #p <0.05, ##p < 0.01, and ###p < 0.001 vs. the N-0 h group, **p < 0.01 and ***p < 0.001 vs. the I-0 h group.

Figure S6. Relative abundances of the most abundant bacterial taxa at genus level during fermentation with ZSSS.

Figure S7. GC-MS spectra of six groups of samples: reference substance (**a**); ZSSS and GAM (**b**); CN group (**c**); N-48 h group (**d**); CI group (**e**); I-48 h group (**f**). 1. Acetic acid; 2. Propionic acid; 3. Isobutyric acid; 4. Butyric acid; 5. Isovaleric acid; 6. Valeric acid; 7. Isocaproic acid.

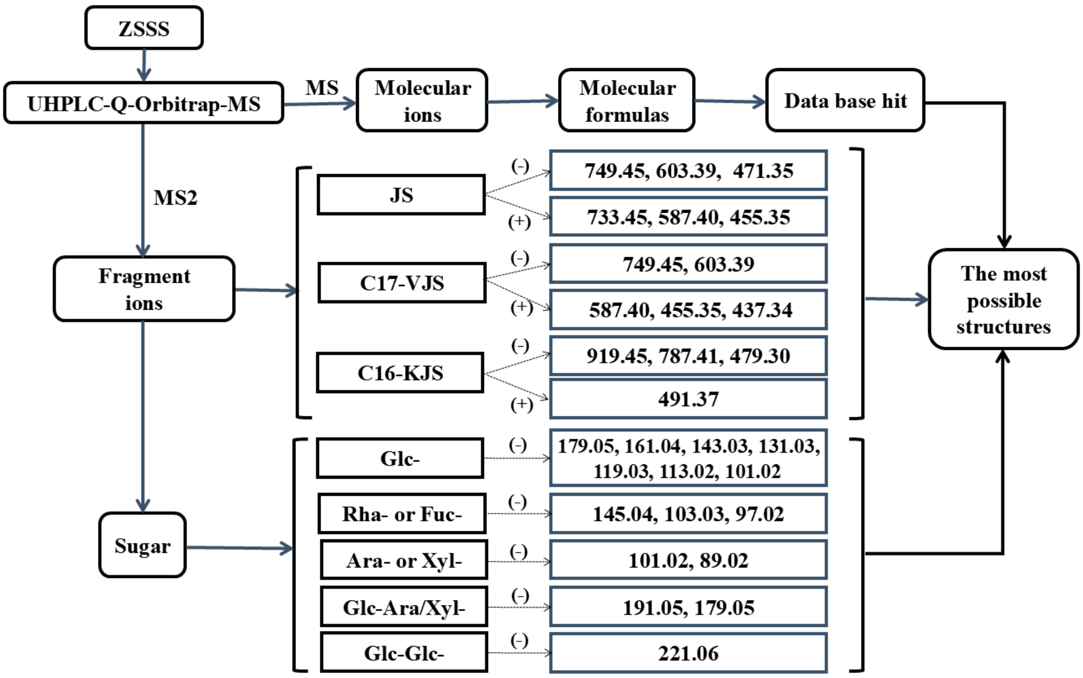
ZSSS
UHPLC-Q-Orbitrap-MS
MS
Molecular ions
Molecular formulas
Data base hit
MS2
Fragment ions
JS
(-)
749.45, 603.39, 471.35
(+)
733.45, 587.40, 455.35
C17-VJS
(-)
749.45, 603.39
(+)
587.40, 455.35, 437.34
C16-KJS
(-)
919.45, 787.41, 479.30
(+)
491.37
Sugar
Glc-
(-)
179.05, 161.04, 143.03, 131.03, 119.03, 113.02, 101.02
Rha- or Fuc-
(-)
145.04, 103.03, 97.02
Ara- or Xyl-
(-)
101.02, 89.02
Glc-Ara/Xyl-
(-)
191.05, 179.05
Glc-Glc-
(-)
221.06
The most possible structures

Figure S1

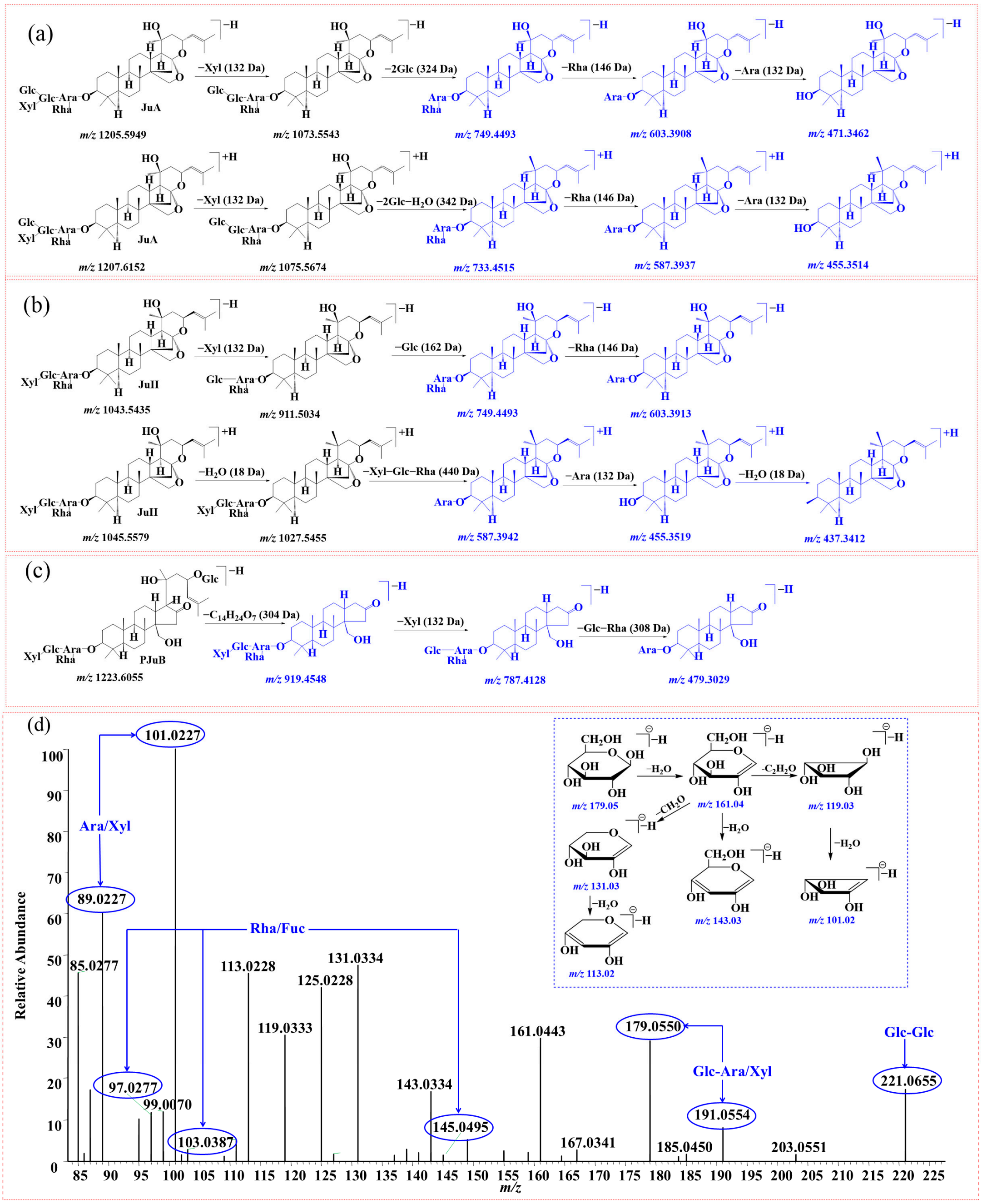

(a)
−Xyl (132 Da)
−2Glc (324 Da)
−Rha (146 Da)
−Ara (132 Da)
JuA
m/z 1205.5949
m/z 1073.5543
m/z 749.4493
m/z 603.3908
m/z 471.3462
−2Glc−H2O (342 Da)
m/z 1207.6152
m/z 1075.5674
m/z 733.4515
m/z 587.3937
m/z 455.3514
(b)
−Glc (162 Da)
JuII
m/z 1043.5435
m/z 911.5034
m/z 749.4493
m/z 603.3913
−H2O (18 Da)
−Xyl−Glc−Rha (440 Da)
m/z 1045.5579
m/z 1027.5455
m/z 587.3942
m/z 455.3519
m/z 437.3412
(c)
PJuB
−C14H24O7 (304 Da)
−Glc−Rha (308 Da)
m/z 1223.6055
m/z 919.4548
m/z 787.4128
m/z 479.3029
(d)
Relative Abundance
m/z
Ara/Xyl
Rha/Fuc
Glc-Ara/Xyl
Glc-Glc
101.0227
89.0227
85.0277
97.0277
99.0070
103.0387
113.0228
119.0333
125.0228
131.0334
143.0334
145.0495
161.0443
167.0341
179.0550
185.0450
191.0554
203.0551
221.0655
m/z 179.05
m/z 161.04
m/z 119.03
m/z 131.03
m/z 143.03
m/z 101.02
m/z 113.02

Figure S2

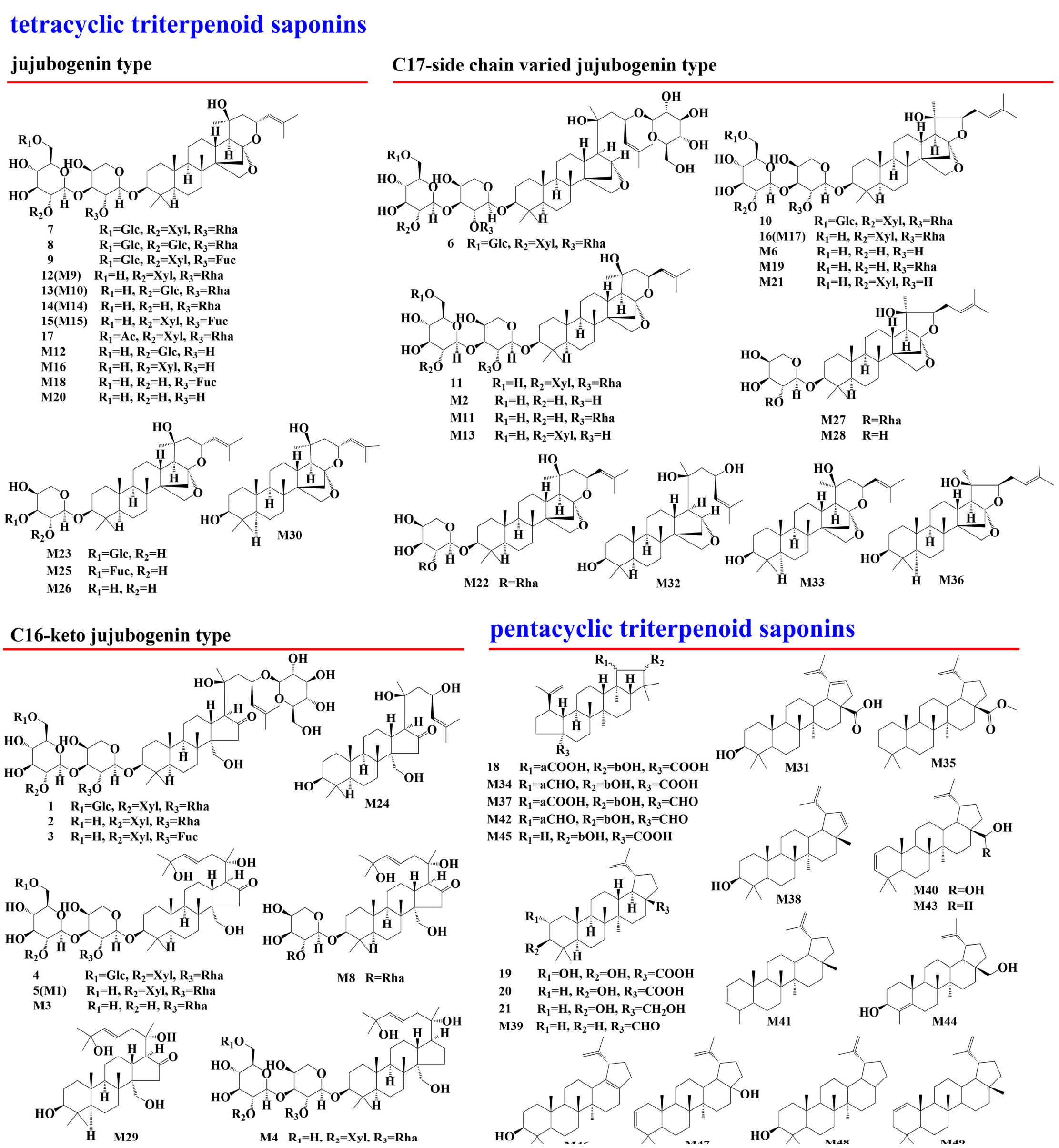
tetracyclic triterpenoid saponins
jujubogenin type
7 R_1=Glc, R_2=Xyl, R_3=Rha
8 R_1=Glc, R_2=Glc, R_3=Rha
9 R_1=Glc, R_2=Xyl, R_3=Fuc
12(M9) R_1=H, R_2=Xyl, R_3=Rha
13(M10) R_1=H, R_2=Glc, R_3=Rha
14(M14) R_1=H, R_2=H, R_3=Rha
15(M15) R_1=H, R_2=Xyl, R_3=Fuc
17 R_1=Ac, R_2=Xyl, R_3=Rha
M12 R_1=H, R_2=Glc, R_3=H
M16 R_1=H, R_2=Xyl, R_3=H
M18 R_1=H, R_2=H, R_3=Fuc
M20 R_1=H, R_2=H, R_3=H
M23 R_1=Glc, R_2=H
M25 R_1=Fuc, R_2=H
M26 R_1=H, R_2=H
M30
C17-side chain varied jujubogenin type
6 R_1=Glc, R_2=Xyl, R_3=Rha
10 R_1=Glc, R_2=Xyl, R_3=Rha
16(M17) R_1=H, R_2=Xyl, R_3=Rha
M6 R_1=H, R_2=H, R_3=H
M19 R_1=H, R_2=H, R_3=Rha
M21 R_1=H, R_2=Xyl, R_3=H
11 R_1=H, R_2=Xyl, R_3=Rha
M2 R_1=H, R_2=H, R_3=H
M11 R_1=H, R_2=H, R_3=Rha
M13 R_1=H, R_2=Xyl, R_3=H
M27 R=Rha
M28 R=H
M22 R=Rha
M32
M33
M36
C16-keto jujubogenin type
1 R_1=Glc, R_2=Xyl, R_3=Rha
2 R_1=H, R_2=Xyl, R_3=Rha
3 R_1=H, R_2=Xyl, R_3=Fuc
M24
4 R_1=Glc, R_2=Xyl, R_3=Rha
5(M1) R_1=H, R_2=Xyl, R_3=Rha
M3 R_1=H, R_2=H, R_3=Rha
M8 R=Rha
M29
M4 R_1=H, R_2=Xyl, R_3=Rha
pentacyclic triterpenoid saponins
18 R_1=aCOOH, R_2=bOH, R_3=COOH
M34 R_1=aCHO, R_2=bOH, R_3=COOH
M37 R_1=aCOOH, R_2=bOH, R_3=CHO
M42 R_1=aCHO, R_2=bOH, R_3=CHO
M45 R_1=H, R_2=bOH, R_3=COOH
M31
M35
M38
M40 R=OH
M43 R=H
19 R_1=OH, R_2=OH, R_3=COOH
20 R_1=H, R_2=OH, R_3=COOH
21 R_1=H, R_2=OH, R_3=CH_2OH
M39 R_1=H, R_2=H, R_3=CHO
M41
M44

Figure S3

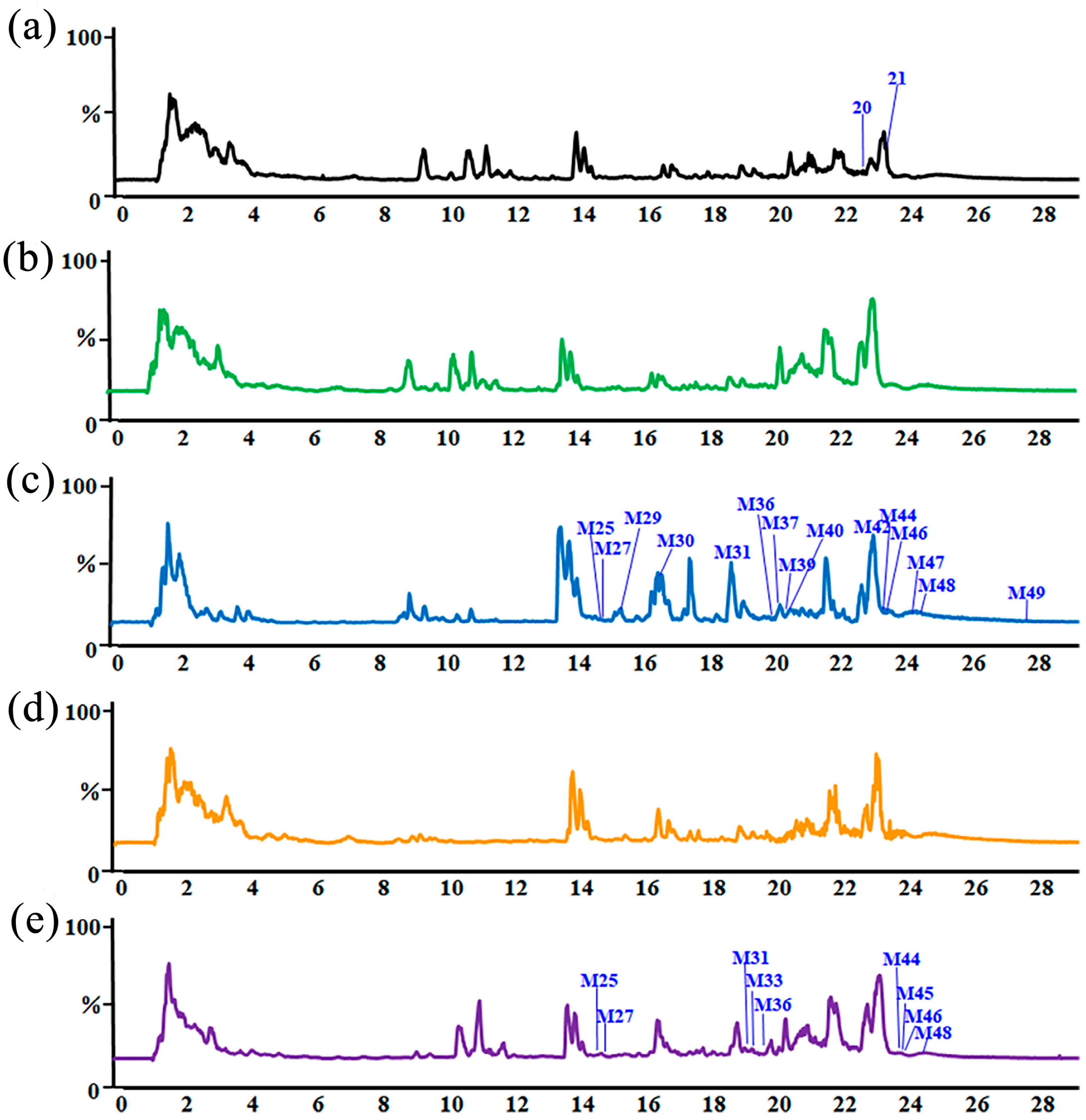
(a)
20
21
(b)
(c)
M25
M27
M29
M30
M31
M36
M37
M39
M40
M42
M44
M46
M47
M48
M49
(d)
(e)
M25
M27
M31
M33
M36
M44
M45
M46
M48
100
%
0
0 2 4 6 8 10 12 14 16 18 20 22 24 26 28

Figure S4

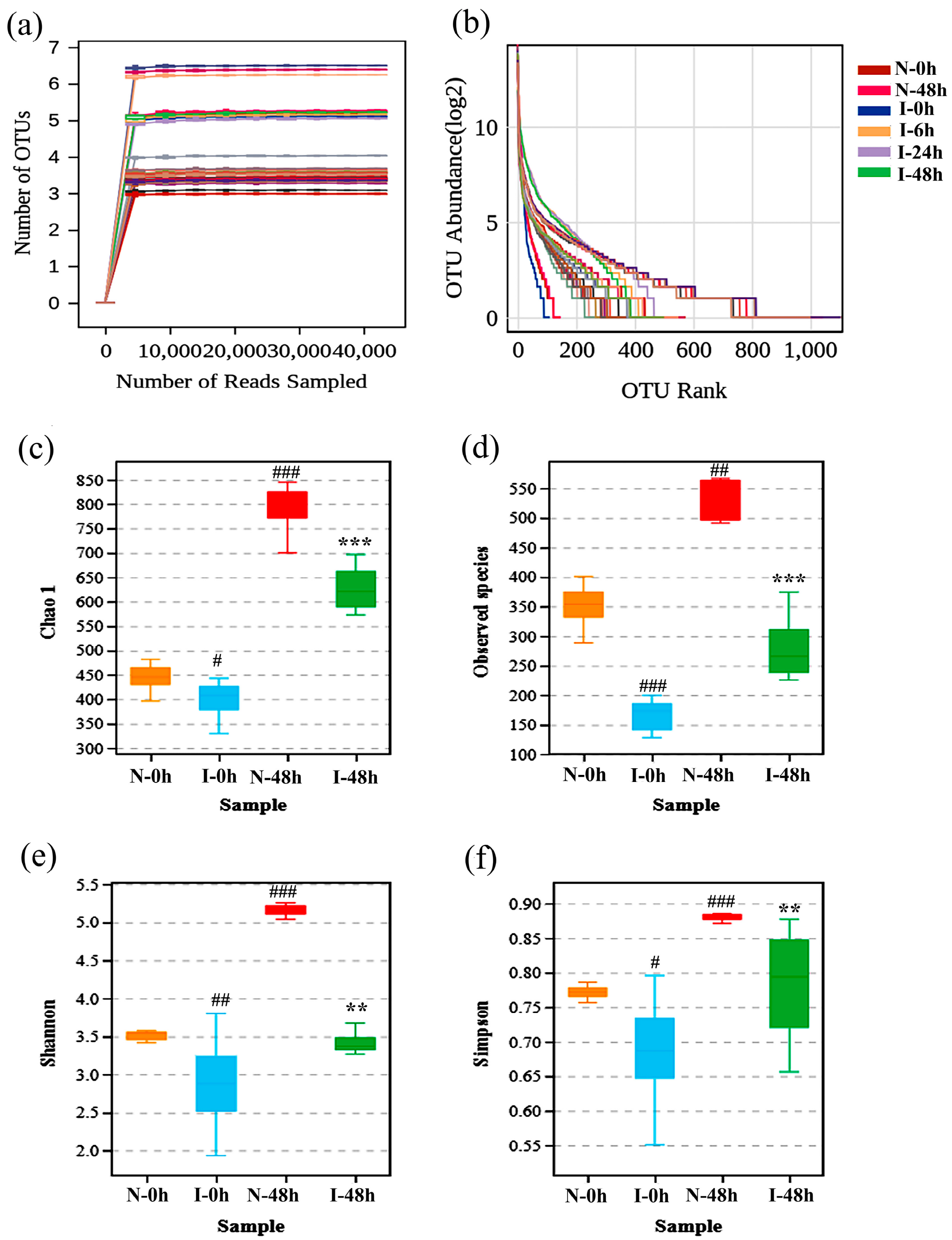

(a)
Number of OTUs
Number of Reads Sampled
0
10,000
20,000
30,000
40,000
(b)
OTU Abundance(log2)
OTU Rank
N-0h
N-48h
I-0h
I-6h
I-24h
I-48h
(c)
Chao 1
Sample
N-0h
I-0h
N-48h
I-48h
(d)
Observed species
Sample
(e)
Shannon
Sample
(f)
Simpson
Sample

Figure S5

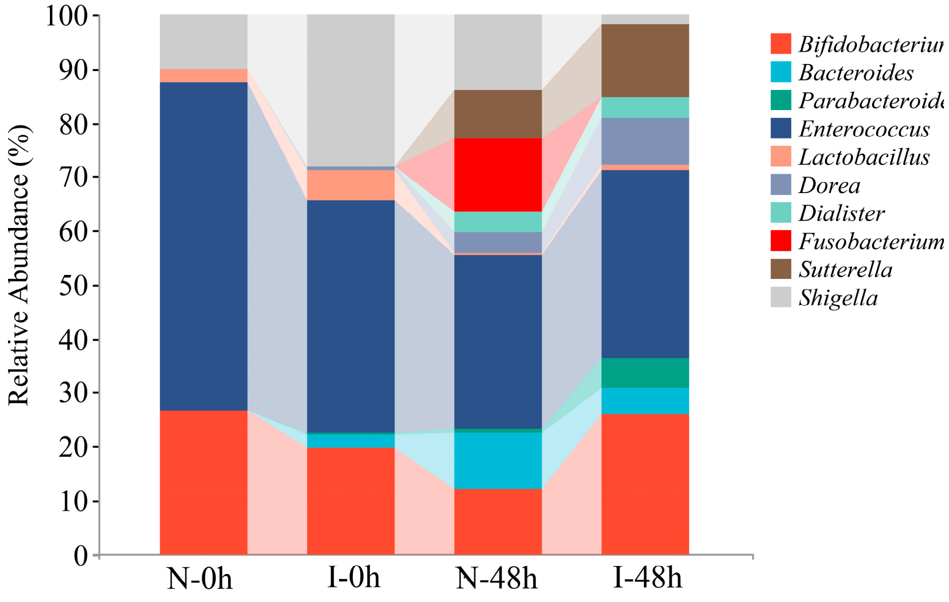
100
90
80
70
60
50
40
30
20
10
0
Relative Abundance (%)
N-0h
I-0h
N-48h
I-48h
Bifidobacteriu
Bacteroides
Parabacteroid
Enterococcus
Lactobacillus
Dorea
Dialister
Fusobacterium
Sutterella
Shigella

Figure S6

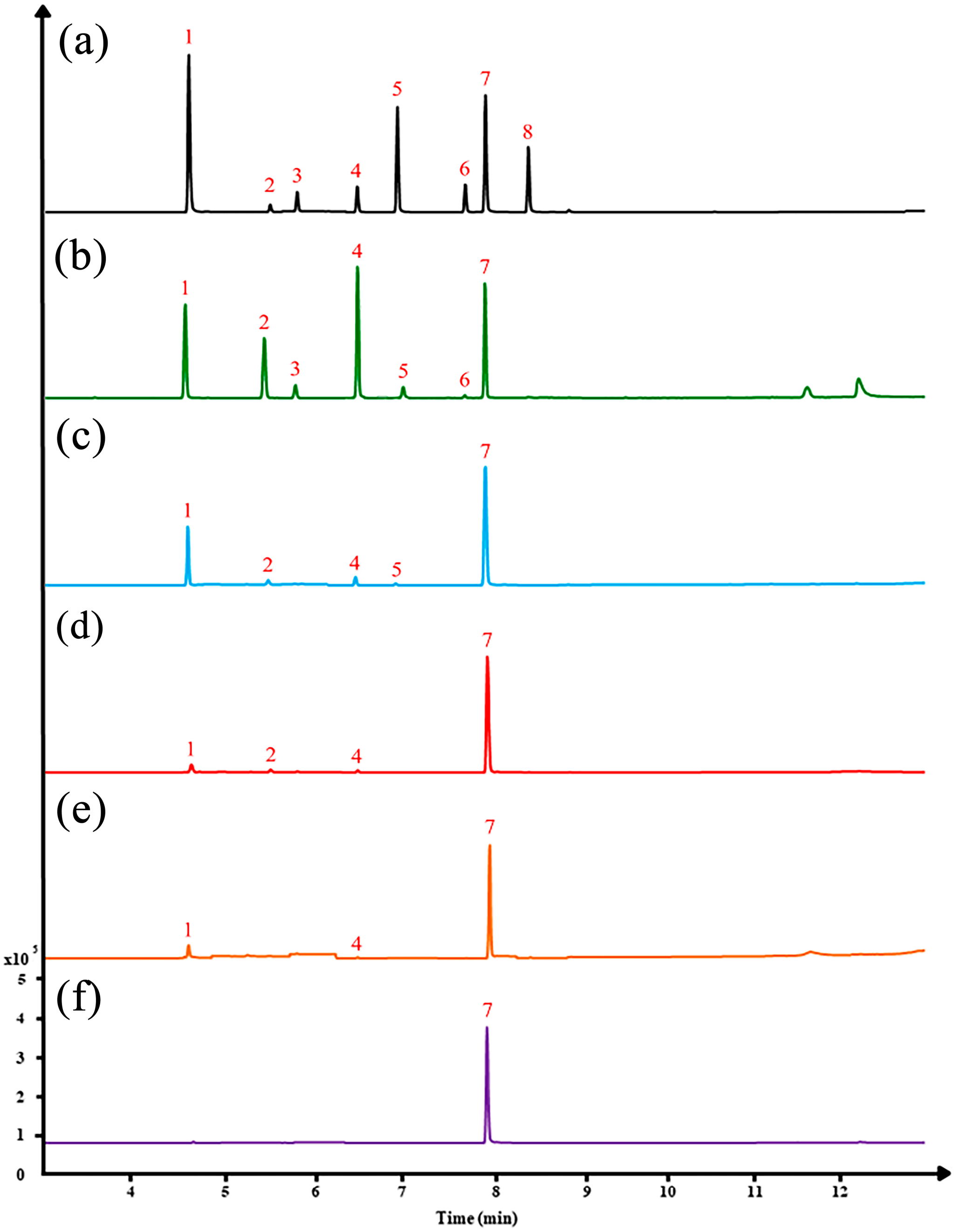

Figure S7

Table S1. Identification of triterpenoid saponins in ZSSS by UPLC-Q-Orbitrap-MS.

No.	t_R/min	Compound	Formula	Select ion	*m/z (*Error/ppm)	Fragment ion
1	2.38	Protojujuboside A (PJuA)	$C_{64}H_{106}O_{32}$	$[M-H]^-$	1385.6594 (−2.26)	1253.6264, 1081.5064, 949.4641, **787.4112**, 625.3592, **479.3014**
				$[M+HCOO]^-$	1431.6649 (−0.99)	1385.6583, 1253.6232, 1081.5064, 949.4636, **787.4137**, 625.3602, **479.3030**
2*	2.94	Protojujuboside B (PJuB)	$C_{58}H_{96}O_{27}$	$[M-H]^-$	1223.6055 (−0.60)	1091.5721, **919.4548**, **787.4128**, 625.3600, **479.3029**, 145.0495, 119.0334, 113.0227, 103.0384, 101.02281
				$[M+HCOO]^-$	1269.6110 (−2.12)	1223.6057, 1091.5566, **919.4546**, **787.4124**, 641.3548, 625.3598, **479.3013**, 179.0548, 161.0445, 143.0337, 131.0336, 119.0335, 113.0229, 101.0227
3	3.60	Protojujuboside B_1 (PJuB_1)	$C_{58}H_{96}O_{27}$	$[M-H]^-$	1223.6055 (−1.80)	1091.5532, **919.4569**, **787.4120**, 641.3540, 625.3597 523.2942, **479.3060**, 179.0549, 161.0444, 145.0492, 143.0336, 131.0334, 119.0334, 113.0229, 103.0385, 101.0227
				$[M+HCOO]^-$	1269.6110 (−1.89)	1223.6064, 1091.5596, 1081.5081, 949.4641, **919.4542**, **787.4133**, 641.3569, **479.2988**, 179.0549, 161.0443, 145.0494, 119.0334, 113.0228, 103.0383, 101.0227
4	4.37	Jujuboside H (JuH)	$C_{58}H_{96}O_{27}$	$[M+HCOO]^-$	1269.6110 (2.76)	1223.6002, 1091.5693, 1073.5517, 991.5122, 945.5023, 927.5000, 765.4437, 619.3856, 455.1415, **221.0659**, 191.0551, 179.0548, 161.0443, 145.0497, 143.0336. 131.0335, 119.0334, 113.0228, 103.0382, 101.0227
5	4.67	Jujuboside G (JuG)	$C_{52}H_{86}O_{22}$	$[M+HCOO]^-$	1107.5581 (−2.71)	1061.5549, 929.5118, 911.5023, **787.4132**, 749.4521, 625.3587, **479.3047**, 161.0443, 145.0493, 131.0336, 101.0227
6	8.00	Jujuboside E (JuE)	$C_{64}H_{106}O_{31}$	$[M-H]^-$	1369.6645 (3.40)	1235.6071, 1205.5877, 1073.5592, 911.5034, **749.4456**, **603.3924**
				$[M+HCOO]^-$	1415.6700 (−3.60)	1369.6532, 1207.6041, 1205.6048, 1073.5513, 911.5000, **749.4493**, **603.3945**
7*	9.21	Jujuboside A (JuA)	$C_{58}H_{94}O_{26}$	$[M+H]^+$	1207.6152 (3.81)	1189.6004, 1075.5674, 1057.5581, 929.5093, 911.4991, **733.4515**, **587.3937**, 473.2532, **455.3514**, 437.3409

No.	t_R/min	Compound	Formula	Select ion	*m/z* (Error/ppm)	Fragment ion
				$[M-H]^-$	1205.5949 (1.58)	1073.5543, 1055.5432, 911.5006, 893.4966, 819.4526, **749.4493**, **603.3908**, **471.3462**, 455.1353, **221.066**2, 191.0552, 179.0550, 161.0443, 145.0477, 143.0335, 131.0335, 119.0319, 113.0213, 103.0374, 101.0214, 97.0264, 89.0214
				$[M+HCOO]^-$	1251.6004 (−1.36)	1205.5976, 1073.5546, 1055.5408, 911.5040, 893.4905, **749.4502**, **603.3895**, **471.3587**, 455.1398, **221.0658**, 191.0559, 179.0550, 161.0443, 145.0483, 143.0338, 131.0335, 119.0334, 113.0228, 103.0371, 101.0215, 97.0266, 89.0216
8	9.30	Jujuboside C (JuC)	$C_{59}H_{96}O_{27}$	$[M+H]^+$	1237.6264 (4.28)	**733.4515**, **587.3937**, 473.2756, **455.3516**, 437.1930
				$[M+HCOO]^-$	1281.6110 (−0.78)	1235.6043, 1089.5459, 1073.5550, 1055.5464, 911.4987, 765.4468, **749.4494**, **603.3915**, **471.3461, 221.0660**, 179.0553, 161.0444, 145.0492, 143.0338, 131.0335, 119.0335, 113.0229, 103.0384, 101.0228, 97.0278, 89.0227
9	10.08	Jujuboside A1 or Jujuboside D (JuA1 or JuD)	$C_{58}H_{94}O_{26}$	$[M+H]^+$	1207.6152 (4.55)	**733.4515**, **587.3937**, 565.3885, 473.2756, **455.3516**, 437.1930
				$[M+HCOO]^-$	1251.6004 (−2.00)	1205.5949, 1073.5553, 1055.5440, 911.5012, 765.4422, **749.4498**, **603.3926,** 455.1387, **221.0660**, 191.0552, 179.0548, 161.0443, 143.0336, 131.0335, 119.0318, 113.0212, 101.0213, 97.0264, 89.0214
10	10.32	Jujuboside IV (JuIV)	$C_{58}H_{94}O_{26}$	$[M+H]^+$	1207.6152 (0.00)	752.4655, 733.9114, 605.4047, **587.3942**, 473.3625, **455.3518**, **437.3412**
				$[M-HCOO]^-$	1251.6004 (−2.57)	1205.5973, 1073.5541, 1055.5407, 927.4913, 911.5007, 893.4914, 765.4378, **749.4492**, **603.3921**, 455.1423, **221.0663**, 191.0555, 179.0550, 161.0444, 143.0338, 131.0335, 119.0335, 113.0229, 103.0381, 101.0228, 97.0278, 89.0227
11	10.61	Jujuboside II (JuII)	$C_{52}H_{84}O_{21}$	$[M+H]^+$	1045.5579 (0.19)	1027.5455, 895.5014, 733.4520, 601.4096, **587.3942**, 473.3626, **455.3519**， **437.3412**

No.	t_R/min	Compound	Formula	Select ion	*m/z (*Error/ppm)	Fragment ion
12*	11.12	Jujuboside B (JuB)	$C_{52}H_{84}O_{21}$	$[M+HCOO]^-$	1089.5476 (−2.75)	1043.5435, 911.5034, 893.4963, 765.4428, **749.4493**, **603.3913**, 161.0443, 143.0342, 131.0336, 119.0337, 113.0228, 103.0386, 101.0227, 89.0227
				$[M+H]^+$	1045.5573 (0.48)	895.5039, 749.3712, **733.4472**, **587.3943**, 473.3626, **455.3518**, 437.1931
				$[M-H]^-$	1043.5421 (0.00)	911.5048, 893.4919, 765.4413, **749.4491**, **603.3909**, **471.3482**, 191.0539, 179.0552, 161.0444, 143.0334, 131.0335, 119.0335, 113.0228, 101.0227, 97.0278, 89.0227
13	11.18	Jujuboside I (JuI)	$C_{53}H_{86}O_{22}$	$[M+HCOO]^-$	1089.5476 (−2.30)	1043.5400, 911.5026, 893.4946, **749.4493**, **603.3910**, **471.3498**, 191.0550, 161.0439, 131.0337, 119.0335, 113.0228, 103.0386, 101.0228, 97.0279, 89.0228
				$[M+H]^+$	1075.5710 (−2.51)	913.5162, **733.4515**, **587.3937**, 541.2370, 473.2756, **455.3516**, 437.1930
				$[M+HCOO]^-$	1119.5581 (−1.34)	1073.5551, 911.5015, 893.4880, 765.4442, **749.4494**, **603.3918**, **471.3457**, **221.0663**, 179.0550, 161.0441, 143.0336, 131.0335, 119.0334, 113.0228, 101.0227, 89.0227
14	11.82	Zizyphus saponin II (Ziz II)	$C_{47}H_{76}O_{17}$	$[M+H]^+$	913.5155 (−0.36)	895.5047, **733.4525**, **587.3943**, 473.3631, **455.3520**, 437.3412
				$[M+HCOO]^-$	957.5053 (−2.61)	911.5029, 765.4446, **749.4503**, **603.3911**, **471.3491**, 161.0443, 131.0333, 119.0333, 103.0383, 101.0228, 97.0274, 89.0227
15*	11.93	Jujuboside B_1 (JuB_1)	$C_{52}H_{84}O_{21}$	$[M+H]^+$	1045.5573 (0.48)	**733.4472**, **587.3943**, 541.2370, 473.2601, **455.3518**, 437.1931
				$[M-H]^-$	1043.5421 (−0.58)	911.5014, 893.4934, 765.4506, **603.3906**, **471.3467**, 191.0550, 179.0535, 161.0440, 143.0326, 131.0336, 119.0334, 113.0229, 103.0381, 101.0228, 97.0272, 89.0227
				$[M+HCOO]^-$	1089.5476 (−2.66)	1043.5478, 911.5022, 893.4957, 765.4450, **749.4497**, **603.3907, 471.3486**, 191.0551, 179.0555, 161.0442, 143.0336, 131.0335, 119.0334, 113.0228, 103.0385, 101.0228, 97.0277, 89.0227
16	12.24	Jujuboside III (JuIII)	$C_{52}H_{84}O_{21}$	[M+H]+	1045.5573 (0.48)	913.5137, 767.4568, 751.4623, 733.4512, 714.6120, **587.3943**, 541.2370, 473.2576, **455.3518**, **437.1931**

No.	t_R/min	Compound	Formula	Select ion	*m/z* (Error/ppm)	Fragment ion
				$[M+HCOO]^-$	1089.5476 (−2.39)	1043.5437, 911.5025, 893.4887, 765.4457, **749.4494**, **603.3920**, 191.0552, 179.0550, 161.0437, 143.0338, 131.0335, 119.0332, 113.0229, 103.03867, 101.0228, 97.0278, 89.0228
17	13.24	Acetyljujuboside B (AcJuB)	$C_{54}H_{86}O_{22}$	$[M+HCOO]^-$	1131.5581 (−2.12)	1085.5516, 1043.5424, 1025.5356, 911.5007, 893.4910, **749.4494**, **603.3911**, **471.3467**
18*	18.15	Ceanothic acid (CA)	$C_{30}H_{46}O_5$	$[M-H]^-$	485.3261 (−3.30)	**423.3275**
19*	19.54	Alphitolic acid (AA)	$C_{30}H_{48}O_4$	$[M-H]^-$	471.3468 (−3.18)	407.3327
20*	22.57	Betulinic acid (BA)	$C_{30}H_{48}O_3$	$[M+H]^+$	457.3676 (0.00)	439.3549, 411.3611, 393.3486, 203.1787, 191.1712
				$[M+HCOO]^-$	502.3750 (−0.18)	316.3350, 154.0294
				$[M-H]^-$	455.3511 (4.17)	409.3436
21*	23.63	Betulin (BE)	$C_{30}H_{50}O_2$	$[M+H]^+$	443.3883 (0.05)	425.2152, 413.2668, 407.3678, 395.2774
				$[M-H]^-$	441.2534 (2.73)	**423.3154**, 271.2271

*Comparison with authentic standards.

Table S2. Metabolites of triterpenoid saponins in Normal and Insomnia Human Intestinal Microbiota Identified by UPLC-Q-Orbitrap-MS.

No.	t_R/min	Identification	Formula	Select ion	*m/z (*Error/ppm)	Fragment ion	Source
M1*	4.67	JuG	$C_{52}H_{86}O_{22}$	$[M+HCOO]^-$	1107.5581 (−2.71)	1061.5549, 929.5118, 911.5023, **787.4132**, 749.4521, 625.3587, **479.3047**, 161.0443, 145.0493, 131.0336, 101.0227	N, I
M2	6.85	JuII−Xyl−Rha	$C_{41}H_{66}O_{13}$	$[M+HCOO]^-$	811.4485 (−0.98)	765.4453, 619.3862, 131.0335, 119.0335, 113.0226, 101.0228, 89.0227	N, I
M3	7.52	JuG−Xyl	$C_{47}H_{78}O_{18}$	$[M+HCOO]^-$	975.5170 (−1.54)	929.5103, 767.4685, 749.4476, 603.3887, 471.3466	N, I
M4	8.87	JuG+2H−O	$C_{52}H_{88}O_{21}$	$[M-H]^-$	1047.5745 (−1.05)	915.5337, 753.4813, 607.4210	I
M5	10.17	JuB+O	$C_{52}H_{84}O_{22}$	$[M+HCOO]^-$	1105.5425 (0.36)	1059.5427, 927.4971, 765.4439, 619.3872, 161.0444, 119.0333, 103.0384, 101.0227	I
M6	10.39	JuIII−Xyl−Rha	$C_{41}H_{66}O_{13}$	$[M+HCOO]^-$	811.4485 (−1.60)	765.4454, **603.3914**	N, I
M7	10.46	JuB+$C_5H_{10}O_5$	$C_{57}H_{92}O_{25}$	$[M+HCOO]^-$	1221.5909 (−0.82)	1175.5766, 1043.5440, 911.5012, 893.5158, 765.4428, **749.4489**, **603.3911**, **471.3512**	N
M8	10.86	JuG−Xyl−Glc	$C_{41}H_{68}O_{13}$	$[M+HCOO]^-$	813.4641 (−1.72)	767.4580, 749.4530, 625.3594, 607.3444, **479.3035**	N, I
M9*	11.12	JuB	$C_{52}H_{84}O_{21}$	$[M+H]^+$	1045.5573 (0.48)	895.5039, 749.3712, **733.4472**, **587.3943**, 473.3626, **455.3518**, 437.1931	N, I
				$[M+HCOO]^-$	1089.5476 (−2.30)	1043.5400, 911.5026, 893.4946, **749.4493**, **603.3910**, **471.3498**, 191.0550, 161.0439, 131.0337, 119.0335, 113.0228, 103.0386, 101.0228, 97.0279, 89.0228	
M10*	11.18	JuI	$C_{53}H_{86}O_{22}$	$[M+H]^+$	1075.5710 (−2.51)	913.5162, **733.4515**, **587.3937**, 541.2370, 473.2756, **455.3516**, 437.1930	N, I
				$[M+HCOO]^-$	1119.5581 (−1.34)	1073.5551, 911.5015, 893.4880, 765.4442, **749.4494**, **603.3918**, **471.3457**, **221.0663**, 179.0550, 161.0441, 143.0336, 131.0335, 119.0334, 113.0228, 101.0227, 89.0227	
M11	11.59	JuII−Xyl	$C_{47}H_{76}O_{17}$	$[M+HCOO]^-$	957.5053 (−2.92)	911.5021, **749.4482**, **603.3865**	N, I
M12	11.67	JuI−Rha	$C_{47}H_{76}O_{18}$	$[M-H]^-$	927.4958 (−1.83)	765.4434, 603.3877	N, I

No.	t_R/min	Identification	Formula	Select ion	*m/z* (Error/ppm)	Fragment ion	Source
				[M+HCOO]$^-$	973.5013 (−0.92)	927.4975, 765.4430, 603.3909, 471.3529	
M13	11.76	JuII−Rha	$C_{46}H_{74}O_{17}$	[M+HCOO]$^-$	943.4908 (0.53)	897.4852, 765.4424, 735.4318, **603.3869**	I
M14*	11.82	Ziz II	$C_{47}H_{76}O_{17}$	[M+H]$^+$	913.5155 (−0.36)	895.5047, **733.4525**, **587.3943**, 473.3631, **455.3520**, 437.3412	N, I
				[M+HCOO]$^-$	957.5053 (−2.61)	911.5029, 765.4446, **749.4503**, **603.3911**, **471.3491**, 161.0443, 131.0333, 119.0333, 103.0383, 101.0228, 97.0274, 89.0227	
M15*	11.93	JuB_1	$C_{52}H_{84}O_{21}$	[M+H]$^+$	1045.5573 (0.48)	**733.4472**, **587.3943**, 541.2370, 473.2601, **455.3518**, 437.1931	N, I
				[M−H]$^-$	1043.5421 (−0.58)	911.5014, 893.4934, 765.4506, **603.3906**, **471.3467**, 191.0550, 179.0535, 161.0440, 143.0326, 131.0336, 119.0334, 113.0229, 103.0381, 101.0228, 97.0272, 89.0227	
				[M+HCOO]$^-$	1089.5476 (−2.66)	1043.5478, 911.5022, 893.4957, 765.4450, **749.4497**, **603.3907,** **471.3486**, 191.0551, 179.0555, 161.0442, 143.0336, 131.0335, 119.0334, 113.0228, 103.0385, 101.0228, 97.0277, 89.0227	
M16	12.12	JuB−Rha/JuB_1−Fuc	$C_{46}H_{74}O_{17}$	[M+HCOO]$^-$	943.4908 (−0.64)	897.4857, 765.4428, **603.3902**	N, I
M17*	12.24	JuIII	$C_{52}H_{84}O_{21}$	[M+H]+	1045.5573 (0.48)	913.5137, 767.4568, 751.4623, 733.4512, 714.6120, **587.3943**, 541.2370, 473.2576, **455.3518**	N, I
				[M+HCOO]$^-$	1089.5476 (−2.39)	1043.5437, 911.5025, 893.4887, 765.4457, **749.4494**, **603.3920**, 191.0552, 179.0550, 161.0437, 143.0338, 131.0335, 119.0332, 113.0229, 103.03867, 101.0228, 97.0278, 89.0228	
M18	12.76	JuB_1−Xyl	$C_{47}H_{76}O_{17}$	[M+HCOO]$^-$	957.5053 (−2.40)	911.5015, 765.4458, **749.4498**, **603.3884**, 143.0334, 131.0335, 119.0337, 113.0228, 101.0228, 89.0227	N, I
M19	12.94	JuIII−Xyl	$C_{47}H_{76}O_{17}$	[M+HCOO]$^-$	957.5053 (−3.55)	911.4998, **749.4510**, **603.3910**, 161.0448, 143.0335, 131.0335, 119.0334, 113.0229, 103.0383, 101.0228, 89.0227	N, I
M20	12.97	Ziz II−Rha (Bacopaside IV)	$C_{41}H_{66}O_{13}$	[M+HCOO]$^-$	811.4485 (−1.11)	765.4423, **603.3895**, 131.0335, 113.0226, 101.0228, 89.0226	N, I

No.	t_R/min	Identification	Formula	Select ion	*m/z* (Error/ppm)	Fragment ion	Source
M21	13.27	JuIII−Rha	$C_{46}H_{74}O_{17}$	$[M+HCOO]^-$	943.4908 (−1.38)	897.5217, 765.4429, **603.3919**, 191.0551, 179.0552, 161.0441, 143.0330, 131.0334, 119.0335, 113.0228, 101.0226, 89.0226	I
M22	13.50	JuII−Xyl−Glc	$C_{41}H_{66}O_{12}$	$[M+HCOO]^-$	795.4536 (−0.88)	**749.4450**	N, I
M23	14.08	Ziz II−Glc/JuB−Rha−Xyl	$C_{41}H_{66}O_{12}$	$[M-H]^-$	749.4481 (2.53)	**603.3896**, 103.0383, 101.0229, 89.0227	N, I
				$[M+HCOO]^-$	795.4536 (−0.75)	**749.4498**, **603.3891,** 145.0493, 103.0383, 101.0228, 89.0228	
M24	14.88	Protojujubogenin	$C_{30}H_{50}O_5$	$[M+H]^+$	**491.3731** (−0.81)	473.3623, 455.3516, 437.3417, 417.2996, 373.2734	N, I
M25	14.96	JuB_1−Xyl−Glc	$C_{41}H_{66}O_{12}$	$[M-H]^-$	749.4481 (−0.40)	**603.3902**, 103.0382, 101.0229, 89.0227	N, I
				$[M+HCOO]^-$	795.4536 (−1.01)	**749.4484**, **603.3891**, **471.3858**, 101.0228, 89.0227	
M26	15.00	JuB_1−Xyl−Glc−Fuc	$C_{35}H_{56}O_8$	$[M+H]^+$	605.4047 (−0.50)	**587.3941**, **455.3520**, 437.3413	N, I
				$[M+Na]^+$	627.3867 (0.80)	605.4054, **587.3936**, **455.3519**, 437.3415	
				$[M+HCOO]^-$	649.3946 (4.18)	631.2596, 613.0322, 485.3260	
M27	15.71	JuIII−Xyl−Glc	$C_{41}H_{66}O_{12}$	$[M+HCOO]^-$	795.4536 (0.25)	**749.4491**, **603.3890**, 145.0493, 103.0385, 101.0228, 89.0226	N, I
M28	15.89	JuIII−Xyl−Glc−Rha	$C_{35}H_{56}O_8$	$[M+H]^+$	605.4047 (−0.17)	587.3938, **455.3930**, **437.3415**	N, I
M29	16.53	JuG−Xyl−Glc−Rha−Ara	$C_{30}H_{50}O_5$	$[M+H]^+$	**491.3731** (0.81)	473.3622, 455.3514, 451.0996, 437.3410, 419.3306	N, I
M30	19.22	Jujubogenin	$C_{30}H_{48}O_4$	$[M+H]^+$	473.3625 (0.85)	**455.3519**, 437.3413, 419.3301, 401.3199	N, I
M31	19.32	BA−H	$C_{30}H_{47}O_3$	$[M+H]^+$	455.3525 (0.18)	437.0432, 409.9337, 391.2309, 247.7850	N, I
M32	19.64	JuE−Glc−Rha−Xyl−Glc−Ara−Glc	$C_{30}H_{50}O_4$	$[M+H]^+$	475.3781 (−0.42)	457.3676, 439.3574, 427.3569, 421.3468	N, I
M33	19.73	JuII−Xyl−Glc−Rha−Ara	$C_{30}H_{48}O_4$	$[M+H]^+$	473.3625 (−0.42)	**455.3519**, **437.3413**, 419.3308	N, I
M34	19.85	CA−O	$C_{30}H_{46}O_4$	$[M-H]^-$	469.3323 (−0.21)	241.2170, 227.0313, 152.9945	N, I
M35	20.09	BA−O+CH_3	$C_{31}H_{51}O_2$	$[M+H]^+$	455.3889 (−0.42)	233.3204, 203.9277, 191.2013, 189.7725	N, I

No.	t_R/min	Identification	Formula	Select ion	*m/z (*Error/ppm)	Fragment ion	Source
M36	20.50	JuIV−Glc−Xyl−Glc−Rha−Ara	$C_{30}H_{48}O_4$	$[M+H]^+$	473.3625 (−0.21)	**455.3519**, **437.3412**, 391.2842, 369.2787	N, I
M37	20.71	CA−O	$C_{30}H_{46}O_4$	$[M-H]^-$	469.3323 (0.11)	379.0840	N, I
M38	20.96	BE−H_2O	$C_{30}H_{48}O$	$[M+H]^+$	425.3777 (−0.59)	207.1473, 203.2765, 189.7351, 175.3850	N
M39	20.96	BA+H−2O	$C_{30}H_{49}O$	$[M+H]^+$	426.3856 (−0.23)	233.2326, 192.0502, 205.0863, 135.9881, 193.2786, 179.1194	I
M40	20.96	BA−H_2O+H	$C_{30}H_{49}O_2$	$[M+H]^+$	441.3732 (−0.34)	423.2153, 233.3348, 135.0446, 203.7725, 191.8164, 189.7021, 177.1573, 175.2460	N, I
M41	23.60	BE−2O−CH_3	$C_{29}H_{46}$	$[M+H]^+$	395.3672 (−0.05)	203.4827, 191.3417, 189.4383, 175.8829	N, I
M42	23.61	CA−2O	$C_{30}H_{46}O_3$	$[M-H]^-$	453.3374 (−0.22)	407.6086, 397.0147	N, I
M43	23.65	BE−H_2O	$C_{30}H_{48}O$	$[M+H]^+$	425.3777 (0.16)	407.3361, 217.7048, 207.8523, 203.1078, 191.3512, 189.5094	N, I
M44	23.83	BE−CH_3	$C_{29}H_{46}O_2$	$[M+H]^+$	427.3570 (0.19)	409.8267, 390.2322, 175.0085, 163.3118	N, I
M45	24.11	CA−COOH	$C_{29}H_{46}O_3$	$[M-H]^-$	441.3374 (2.04)	378.9474	N
M46	24.82	BA−HCOOH	$C_{29}H_{47}O$	$[M+H]^+$	411.3626 (−0.10)	423.2153, 233.3348, 135.0446, 203.7725, 191.8164, 189.7021, 177.1573, 175.2460	N, I
M47	25.05	BA−H_2O−CO	$C_{29}H_{47}O$	$[M+H]^+$	411.3626 (−0.27)	221.1238, 191.2013, 189.9171, 203.8544, 135.7212	N, I
M48	28.22	BE−CH_2O	$C_{29}H_{48}O$	$[M+H]^+$	413.2677 (−0.22)	203.2033, 191.5581, 189.3414, 175.5103	I
M49	28.22	BE−2O−CH_3	$C_{29}H_{46}$	$[M+H]^+$	395.36729 (0.05)	203.1984, 191.2841, 189.5653, 175.2950	N, I

N: normal human gut microbiota sample, I: Insomnia human gut microbiota sample.

*The compound that is both a prototype component and a metabolite.

Table S3. Comparison of Metabolites Source and Biotransformation Reaction of ZSSS Extract in Insomnia Human Intestinal Microbiota.

No.	Source		Reaction	No.	Source		Reaction
	Normal	Insomnia			Normal	Insomnia	
M1	▲	▲	deglycosylation	M26	▲	▲	deglycosylation
M2	▲	▲	deglycosylation	M27	▲	▲	deglycosylation
M3	▲	▲	deglycosylation	M28	▲	▲	deglycosylation
M4	-	▲	deoxygenation	M29	▲	▲	deglycosylation
M5	-	▲	oxidation	M30	▲	▲	deglycosylation
M6	▲	▲	deglycosylation	M31	▲	▲	dehydrogenation
M7	▲	-	glycosylation	M32	▲	▲	deglycosylation
M8	▲	▲	deglycosylation	M33	▲	▲	deglycosylation
M9	▲	▲	deacylation, deglycosylation	M34	▲	▲	deoxygenation
M10	▲	▲	deglycosylation	M35	▲	▲	deoxygenation, methylation
M11	▲	▲	deglycosylation	M36	▲	▲	deglycosylation
M12	▲	▲	deglycosylation	M37	▲	▲	deoxygenation
M13	-	▲	deglycosylation	M38	▲	-	dehydration
M14	▲	▲	deglycosylation	M39	-	▲	hydrogenation, deoxygenation

No.	Source		Reaction	No.	Source		Reaction
	Normal	Insomnia			Normal	Insomnia	
M15	▲	▲	deglycosylation	M40	▲	▲	dehydration, hydrogenation
M16	▲	▲	deglycosylation	M41	▲	▲	demethylation, deoxygenation
M17	▲	▲	deglycosylation	M42	▲	▲	deoxygenation
M18	▲	▲	deglycosylation	M43	▲	▲	dehydration
M19	▲	▲	deglycosylation	M44	▲	▲	demethylation
M20	▲	▲	deglycosylation	M45	▲	-	decarboxylation
M21	-	▲	deglycosylation	M46	▲	▲	decarboxylation
M22	▲	▲	deglycosylation	M47	▲	▲	dehydration, Loss of CO
M23	▲	▲	deglycosylation	M48	-	▲	dehydroxylation (Loss of CH_2O)
M24	▲	▲	deglycosylation	M49	▲	▲	demethylation, deoxygenation
M25	▲	▲	deglycosylation				

▲: detected; -: undetected.

Table S4. Retention time and characteristic ions of seven SCFAs compounds.

Compounds	Molecular weight	Retention time	Qualitative Ion
Acetic acid	60	4.67	42、45、60
Propionic acid	74	5.56	73、74、45、43、32、31
Isobutyric acid	88	5.85	43、73、88、55、41、39
Butyric acid	88	6.51	60、73、41、42、43、45
Isovaleric acid	106	6.96	74、87、41、73、56、45
Valeric acid	102	7.68	60、73、44、41、55、43
2-Ethylbutyric acid	116	7.91	88、73、43、55、87、41
Isocaproic acid	116	8.38	73、74、55、60、43、41、39、45、59、83

Table S5. Calibration curves, Linearity range, precision, repeatability and stability of seven SCFAs determined using GC-MS. (*n* = 6).

Compounds	Calibration curves	Linear range ($mmol*L^{-1}$)	r^2	Inter-Day Precision (RSD%)	Intra-Day Precision (RSD%)	Repeatability (RSD%)	Stability (RSD%)
Acetic acid	Y = 0.2582 x + 0.2394	0.50-30	0.9904	2.70	2.50	4.96	2.60
Propionic acid	Y = 0.2326 x + 0.0033	0.06-5	0.9961	2.44	3.03	4.40	2.22
Isobutyric acid	Y = 0.3377 x − 0.0109	0.03-3	0.9993	2.53	2.62	4.53	3.75
Butyric acid	Y = 0.4481 x − 0.0071	0.02-5	0.9979	1.92	2.78	3.32	2.68
Isovaleric acid	Y = 0.1989 x + 0.0022	0.08-5	0.9972	2.04	2.21	1.81	3.60
Valeric acid	Y = 0.3645 x − 0.0085	0.04-3	0.9998	1.49	2.95	4.15	3.41
Isocaproic acid	Y = 2.6743 x + 0.0014	0.006-1.2	0.9998	4.58	4.72	3.26	3.58

Table S6. The relative peak area percentage and docking results of each prototype component and metabolite in normal and insomnia human intestinal microbiota. -: Docking unsuccessful.

No	Compound	0 h		6 h		12 h		24 h		48 h		$GABA_A$		5-HT	
		N	I	N	I	N	I	N	I	N	I	Binging energy	Binding site	Binging energy	Binding site
1	PJuA	0.10	0.15	0.09	0.16	0.08	0.14	0.07	0.13	0.00	0.08	-	-	-	-
2	PJuB	0.11	0.14	0.07	0.16	0.07	0.15	0.05	0.14	0.00	0.10	-	-	-	-
3	$PJuB_1$	0.09	0.17	0.09	0.16	0.06	0.15	0.05	0.14	0.00	0.09	-	-	-	-
4	JuH	0.15	0.13	0.14	0.14	0.10	0.12	0.00	0.12	0.00	0.11	-	-	-	-
5 (M1)	JuG	0.18	0.22	0.17	0.00	0.14	0.00	0.12	0.01	0.14	0.01	0.42	D: GLN204	605.48	TYR370
6	JuE	0.11	0.11	0.10	0.12	0.09	0.17	0.09	0.21	0.00	0.00	-	-	-	-
7	JuA	0.11	0.19	0.10	0.46	0.04	0.06	0.00	0.03	0.00	0.00	−1.61	C: VAL190, C: GLU189	604.03	TYR370; SER159; ASP155
8	JuC	0.09	0.23	0.06	0.34	0.06	0.16	0.00	0.07	0.00	0.00	11.66	C: TYR58, C: SER195	928.41	ASN343
9	JuA_1 or JuD	0.09	0.54	0.06	0.19	0.00	0.13	0.00	0.00	0.00	0.00	3.25	C: ASN60, D: LYS156	781.61	THR160; SER242; TRP151
10	JuIV	0.20	0.07	0.23	0.05	0.19	0.03	0.19	0.03	0.00	0.00	-	-	1385	SER242
11	JuII	0.20	0.01	0.21	0.01	0.18	0.01	0.19	0.01	0.19	0.01	−4.99	D: GLN204	294.61	PHE234; SER242
12 (M9)	JuB	0.12	0.11	0.07	0.12	0.09	0.11	0.08	0.11	0.07	0.10	−2.55	C: ASN60, D: LYS156, D: ILE202	796.25	SER159
13 (M10)	JuI	0.07	0.20	0.07	0.20	0.03	0.15	0.00	0.14	0.00	0.15	−3.45	D: GLN204	636.55	SER242

No	Compound	0 h		6 h		12 h		24 h		48 h		$GABA_A$		5-HT	
		N	I	N	I	N	I	N	I	N	I	Binging energy	Binding site	Binging energy	Binding site
14 (M14)	Ziz II	0.02	0.13	0.05	0.13	0.04	0.19	0.03	0.25	0.02	0.13	−3.8	C: ASN60, D: LYS156, D: GLN204	169	THR160; SER242
15 (M15)	JuB_1	0.04	0.13	0.06	0.13	0.09	0.14	0.09	0.11	0.06	0.16	−2.51	D: GLN204, D: ILE202	218.73	SER242
16 (M17)	JuIII	0.18	0.16	0.18	0.03	0.03	0.03	0.16	0.04	0.16	0.03	−2.78	C: ASP192	611.78	ASP155
17	AcJuB	0.01	0.15	0.17	0.05	0.17	0.04	0.17	0.03	0.00	0.22	-	-	408.85	THR160; SER242
18	CA	0.10	0.16	0.08	0.17	0.05	0.13	0.04	0.14	0.06	0.08	-	-	-	-
19	AA	0.07	0.12	0.06	0.12	0.07	0.14	0.06	0.15	0.07	0.15	-	-	-	-
20	BA	0.78	0.03	0.01	0.03	0.02	0.02	0.02	0.03	0.02	0.04	−7.78	D: LYS156	-	-
21	BE	0.00	0.28	0.00	0.28	0.00	0.00	0.22	0.00	0.22	0.00	-	-	-	-
M2	JuII−Xyl−Rha	0.00	0.17	0.00	0.19	0.11	0.17	0.10	0.15	0.11	0.00	−7.72	C: ASP192	76.45	SER242
M3	JuG−Xyl	0.11	0.16	0.11	0.14	0.10	0.15	0.10	0.13	0.00	0.00	−2.28	C: SER195, C: TYR58, D:LYS156	353.9	TYR370
M4	JuG+2H−O	0.00	0.26	0.00	0.18	0.00	0.23	0.00	0.18	0.14	0.00	−3.48	C: ASN60, C: SER195, C: ASP192, D: HIS102	478.92	ILE206; ASP155; ILE152
M20	Ziz II−Rha (Bacopaside IV)	0.00	0.00	0.53	0.00	0.02	0.00	0.05	0.00	0.39	0.00	−5.9	C: ASP192	68.08	SER159
M5	JuB+O	0.00	0.00	0.00	0.39	0.00	0.38	0.00	0.00	0.23	0.00	-	-	-	-

No	Compound	0 h		6 h		12 h		24 h		48 h		GABA$_A$		5-HT	
		N	I	N	I	N	I	N	I	N	I	Binging energy	Binding site	Binging energy	Binding site
M2	JuIII−Xyl−Rha	0.00	0.00	0.20	0.19	0.14	0.18	0.14	0.00	0.14	0.00	−7.29	C: ASP192	103.47	ILE210; SER242
M7	JuB+$C_5H_{10}O_5$	0.00	0.40	0.00	0.30	0.00	0.30	0.00	0.00	0.00	0.00	-	-	-	-
M8	JuG−Xyl−Glc	0.00	0.11	0.13	0.11	0.12	0.13	0.11	0.14	0.00	0.14	−5.52	C: ASN60, C: GLU189, D: GLN204, D: LYS156	108.65	ILE206; SER159
M11	JuII−Xyl	0.00	0.00	0.00	0.22	0.17	0.25	0.18	0.00	0.17	0.00	−4	D: GLN204	175.93	SER239
M12	JuI−Rha	0.00	0.00	0.00	0.01	0.25	0.30	0.21	0.00	0.23	0.00	−6.37	C: GLU189	202.03	SER242
M13	JuII−Rha	0.00	0.01	0.00	0.01	0.00	0.01	0.47	0.01	0.49	0.00	−7.37	C: TYR58	435.54	SER242
M16	JuB−Rha/JuB$_1$−Fuc	0.00	0.03	0.00	0.06	0.00	0.07	0.00	0.07	0.73	0.05	−5.39	C: GLU189	134.53	SER159
M18	JuB$_1$−Xyl	0.00	0.18	0.13	0.18	0.13	0.15	0.12	0.00	0.12	0.00	−3.92	C: ASN60, D: LYS156, D: GLN204	111.74	SER159
M19	JuIII−Xyl	0.00	0.00	0.22	0.01	0.24	0.01	0.23	0.01	0.26	0.02	−4.69	C: SER195, D: LYS156	131.66	ILE206; SER159
M21	JuIII−Rha	0.00	0.17	0.00	0.22	0.00	0.19	0.00	0.20	0.00	0.21	−6.64	C: ASP192	230.13	SER242
M22	JuII−Xyl−Glc	0.00	0.00	0.00	0.31	0.00	0.29	0.20	0.00	0.20	0.00	−8.45	D: ILE202, D: GLN204	56.93	SER242
M23	Ziz II−Glc/JuB−Rha−Xyl	0.00	0.06	0.00	0.11	0.00	0.12	0.15	0.12	0.30	0.13	−6.68	C: THR142	86.58	SER242
M24	Protojujubogenin	0.00	0.05	0.14	0.13	0.15	0.13	0.14	0.13	0.00	0.13	−7.35	D: SER205	0.97	ASP155
M25	JuB$_1$−Xyl−Glc	0.00	0.21	0.00	0.23	0.00	0.18	0.01	0.17	0.01	0.18	−7.49	D: SER205	70.51	SER159

No	Compound	0 h		6 h		12 h		24 h		48 h		$GABA_A$		5-HT	
		N	I	N	I	N	I	N	I	N	I	Binging energy	Binding site	Binging energy	Binding site
M26	JuB_1−Xyl−Glc−Fuc	0.00	0.00	0.00	0.16	0.00	0.16	0.18	0.17	0.24	0.08	−8.17	C: ASN60,D:GLN204	18.98	SER242
M27	JuIII−Xyl−Glc	0.00	0.00	0.00	0.00	0.18	0.00	0.18	0.22	0.20	0.22	−8.35	D: LYS156, C: TYR58	53.72	PHE234
M28	JuIII−Xyl−Glc−Rha	0.00	0.00	0.00	0.00	0.11	0.00	0.13	0.24	0.24	0.28	−8.38	D: LYS156	21.11	GLY238; SER242
M29	JuG−Xyl−Glc−Rha−Ara	0.00	0.00	0.00	0.00	0.32	0.00	0.33	0.00	0.00	0.35	−6.4	C: ASP192, C:PHE77, D: HIS102	2.52	VAL235
M30	Jujubogenin	0.00	0.00	0.00	0.00	0.00	0.14	0.23	0.14	0.35	0.15	−8.83	D: LYS156, D: SER205	-	-
M31	BA−H	0.00	0.00	0.00	0.40	0.00	0.00	0.00	0.00	0.60	0.00	−5.84	C: GLU189	-	-
M32	JuE−Glc−Rha−Xyl−Glc−Ara−Glc	0.00	0.00	0.00	0.44	0.00	0.00	0.00	0.00	0.56	0.00	−9.35	D: SER205	−7.59	THR160
M33	JuII−Xyl−Glc−Rha−Ara	0.00	0.00	0.00	0.38	0.00	0.00	0.12	0.00	0.50	0.00	−9.8	D: SER205	3.32	THR160
M34	CA−O	0.00	0.00	0.00	0.55	0.00	0.00	0.00	0.00	0.45	0.00	−9.76	C: TYR58	2.56	ASP155
M35	BA−O+CH_3	0.00	0.00	0.00	0.36	0.00	0.00	0.31	0.00	0.33	0.00	−8.67	D: LYS156	-	-
M36	JuIV−Glc−Xyl−Glc−Rha−Ara	0.00	0.00	0.00	0.29	0.17	0.00	0.20	0.00	0.35	0.00	−8.66	D: LYS156	-	-
M37	CA−O	0.00	0.00	0.00	0.38	0.21	0.00	0.19	0.00	0.23	0.00	−8.34	D: LYS156	-	-
M38	BE−H_2O	0.00	0.00	0.00	0.28	0.25	0.00	0.24	0.00	0.23	0.00	-	-	-	-

No	Compound	0 h		6 h		12 h		24 h		48 h		$GABA_A$		5-HT	
		N	I	N	I	N	I	N	I	N	I	Binging energy	Binding site	Binging energy	Binding site
M39	BA+H−2O	0.00	0.00	0.00	0.00	0.00	0.00	1.00	0.00	0.00	0.00	−8.67	D: LYS156	-	-
M40	BA−H_2O+H	0.00	0.00	0.00	0.00	0.00	0.00	0.00	0.00	1.00	0.00	−8.42	D: LYS156	-	-
M41	BE−2O−CH_3	0.16	0.19	0.00	0.19	0.16	0.00	0.15	0.00	0.15	0.00	-	-	-	-
M42	CA−2O	0.00	0.50	0.00	0.48	0.00	0.00	0.01	0.00	0.01	0.00	−9.87	C: TYR58	-	-
M43	BE−H_2O	0.00	0.29	0.00	0.28	0.00	0.00	0.21	0.00	0.22	0.00	−8.79	D: LYS160	-	-
M44	BE−CH_3	0.00	0.30	0.00	0.23	0.16	0.00	0.16	0.00	0.15	0.00	−8.98	D: LYS156	-	-
M45	CA−COOH	0.00	0.29	0.00	0.32	0.00	0.00	0.39	0.00	0.00	0.00	−9.46	D: LYS156	−3.25	ASP155; TYR370
M46	BA−HCOOH	0.00	0.13	0.00	0.13	0.25	0.00	0.25	0.00	0.24	0.00	-	-	-	-
M47	BA−H_2O−CO	0.00	0.17	0.00	0.16	0.23	0.00	0.22	0.00	0.22	0.00	−8.25	C:ASN60; D: LYS156	-	-
M48	BE−CH_2O	0.00	0.44	0.00	0.00	0.00	0.00	0.56	0.00	0.00	0.00	−9.83	D: LYS156	-	-
M49	BE−2O−CH_3	0.00	0.00	0.00	0.00	0.00	0.00	0.00	0.00	1.00	0.00	-	-	-	-

Table S7. The information of normal humans and insomnia patients.

Group	No.	Age	Drug	Duration of insomnia	Antibiotics	Diet
Normal	N-1	29	-	-	-	light diet
	N-2	28	-	-	-	light diet
	N-3	55	-	-	-	light diet
	N-4	40	-	-	-	light diet
	N-5	26	-	-	-	light diet
	N-6	34	-	-	-	light diet
Insomnia	I-1	53	-	10	-	light diet
	I-2	28	-	2	-	light diet
	I-3	35	-	7	-	light diet
	I-4	26	-	2	-	light diet
	I-5	20	-	2	-	light diet
	I-6	37	-	5	-	light diet

Table S8. The information of grouping and drug administration of mice.

Group	Number	Drug Name	Dose (g/kg)	Route of Administration	Duration (Days)
CON	12	Normal saline	-	oral gavage	7
DZP	12	diazepam	2	oral gavage	7
ZSSSL	12	ZSSS	0.18	oral gavage	7
ZSSSH	12	ZSSS	0.36	oral gavage	7
ZSSST	12	ZSSST	0.14	oral gavage	7
ZSSSTH	12	ZSSST	0.28	oral gavage	7
